# A Simulation on Potential Secondary Spread of Novel Coronavirus in an Exported Country Using a Stochastic Epidemic SEIR Model

**DOI:** 10.3390/jcm9040944

**Published:** 2020-03-30

**Authors:** Kentaro Iwata, Chisato Miyakoshi

**Affiliations:** 1 Division of Infectious Diseases, Kobe University Hospital, Kobe 650-0017, Japan; 2 Department of Research Support, Center for Clinical Research and Innovation, Kobe City Medical Center General Hospital, Kobe 650-0047, Japan; miyakoshi@wisdomsprout.com

**Keywords:** novel coronavirus, SEIR model, outside China

## Abstract

Ongoing outbreak of pneumonia caused by novel coronavirus (2019-nCoV) began in December 2019 in Wuhan, China, and the number of new patients continues to increase. Even though it began to spread to many other parts of the world, such as other Asian countries, the Americas, Europe, and the Middle East, the impact of secondary outbreaks caused by exported cases outside China remains unclear. We conducted simulations to estimate the impact of potential secondary outbreaks in a community outside China. Simulations using stochastic SEIR model were conducted, assuming one patient was imported to a community. Among 45 possible scenarios we prepared, the worst scenario resulted in the total number of persons recovered or removed to be 997 (95% CrI 990–1000) at day 100 and a maximum number of symptomatic infectious patients per day of 335 (95% CrI 232–478). Calculated mean basic reproductive number (R_0_) was 6.5 (Interquartile range, IQR 5.6–7.2). However, better case scenarios with different parameters led to no secondary cases. Altering parameters, especially time to hospital visit. could change the impact of a secondary outbreak. With these multiple scenarios with different parameters, healthcare professionals might be able to better prepare for this viral infection.

## 1. Introduction

An outbreak of pneumonia caused by novel coronavirus (2019-nCoV) began in December 2019 in Wuhan, China, and the number of the newly reported cases continues to increase. More than 90,000 cases were reported globally, and about 3000 of them have died as of this writing (4 March 2020) [1]. Initially, this virus was believed to be transmitted from some wild animals to humans, and human to human transmission was limited or nonexistent [2], but it turned out that human to human transmission indeed occurs, resulting in thousands of patients in China. As of this writing, more than 10,000 confirmed cases were reported outside of mainland China, found in 72 different countries [1]. However, the impact of secondary transmissions occurring by importing an infected person remains unclear, and might be different depending on the health care system or environment each country has [3,4,5,6,7,8].

While we do not know the extent of transmissibility of this virus precisely, and do not know how serious these impacts of secondary human to human transmission outside China could be, we developed a mathematical model and simulated the possibility and the risk of secondary outbreak caused by 2019-nCoV outside China, in a developed country with a relatively good infectious diseases reporting system and infection prevention practices.

## 2. Experimental Section

We hypothesized a scenario of one person with 2019-CoV infection entering into a community with a population of 1000. The SEIR model was used, with compartment S, E, I, R standing for susceptible, exposed, infected, and recovered or removed respectively. The model describing the status of each compartment is shown in the following differential equations.

(1) $$\frac{dS\left(t\right)}{dt}=-\frac{\beta S\left(t\right)I\left(t\right)}{N}$$

(2) $$\frac{dE\left(t\right)}{dt}=\frac{\beta S\left(t\right)I\left(t\right)}{N}-\delta E\left(t\right)$$

(3) $$\frac{dI\left(t\right)}{dt}=\delta E\left(t\right)-\nu I\left(t\right)$$

(4) $$\frac{dR\left(t\right)}{dt}=vI\left(t\right)$$

where β is force of infection, or disease transmission rate, δ is inverse of latent period (days), and ν is inverse of infectious period (days), or removal rate. Because parameters in developed countries outside China remain unknown as of the current writing, we prepared a combination of hypothetical parameters to simulate multiple scenarios likely or possible to occur. The following are the combination of parameters.

β: (1) 0.01–0.02, (2)0.04–0.06, (3)0.1–0.2, (4)0.4–0.6, (5)0.8–1

δ: (i) 1/4–1/2, (ii) 1/10–1/4, (iii) 1/14–1/10

ν: (a)1/2–1, (b) 1/7–1/2, (c) 1/10–1/7

With five scenarios in β, three in δ, and three in ν, we developed 45 scenarios and simulated each. With stochastic imputation, data between upper limit and lower limit of each parameter were assigned randomly. While certain parameters such as ν outside China remain unknown, we incorporated some known parameters with some intervals into our simulation model. For example, β in a study conducted in China was 1/7 [9], and median incubation period estimated recently was 5.1 days with most developed symptoms by 14 days [10].

N is population size of the community, 1000 in our simulation. Susceptible individuals in class S in contact with the virus enter the exposed class E. Exposed individuals undergo an incubation period ranging from 2 to 14 days, before progressing to the infectious class I: infectious individuals eventually move to R (death or recovered). We assumed no further transmission would occur once a person entered into the status of R. We began the simulation assuming the aggregate of S, E, I, R to be 1000, and simulation started with S = 999, E = 1, I = 0, and R = 0. We constructed mathematical models of prediction for the following 100 days after importation of one infection as follows: we assumed the parameters to be distributed uniformly, which means each parameter has an equal chance of taking a value within the range in each scenario. For example, in scenario (a), the nu was chosen from 0.5 to 1 with equal probability density.

We obtained the number of patients in each status (i.e., S, E, I, and R) at every 0.2 day from the beginning to the 100th day using the Markov Chain Monte Carlo (MCMC) method. We set a sampling sequence of 200 random samples without warmup. Two-sided 95% credible intervals (CrI) were also calculated.

Basic reproductive number (R_0_) was calculated as β/ν by using mean number of 1000 imputations under the condition of each scenario. Analyses were performed with the use of R software (R Foundation for Statistical Computing).

## 3. Results

**Figure 1:**
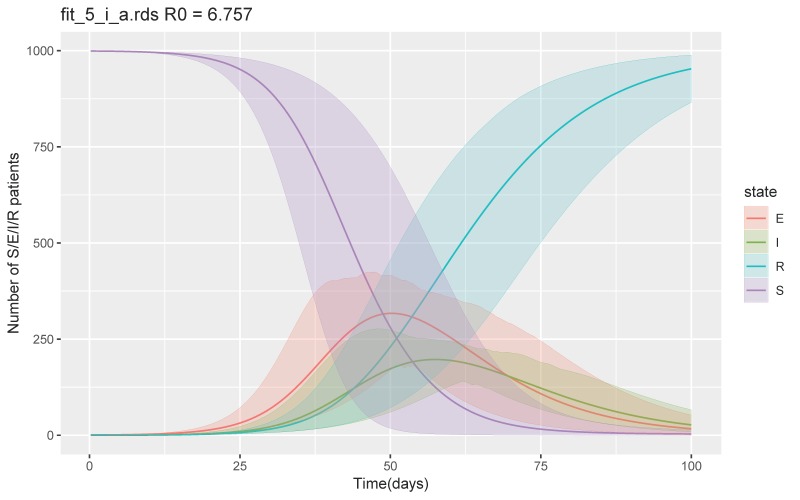
SEIR model with the worst case scenario. Abbreviations S = susceptible E = exposed, I = infected, and R = recovered and removed.

Among 45 scenarios, the worst scenario, in which the largest number of infected and recovered would occur, was when β was 0.8–1, δ was 1/14–1/10, and ν was 1/10–1/7. The simulation showed a total number of recovered or removed patients of 997 (95% CrI 990–1000) at day 100 and maximum number of symptomatic infectious patients per day of 335 (95% CrI 232–478) (Figure 1). Calculated mean basic reproductive number (R_0_) was 6.5 (Interquartile range, IQR 5.6–7.2).

**Figure 2:**
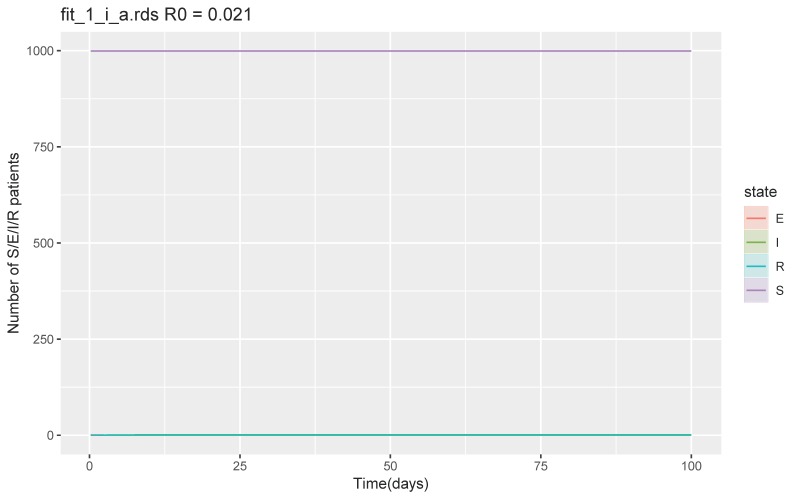
SEIR model with the best-case scenario. Abbreviations S = susceptible, E = exposed, I = infected, and R = recovered and removed.

With the best-case scenario where β was 0.01–0.02, δ was 1/4–1/2, and ν was 1/2–1, a secondary outbreak did not occur, and the total number of recovered or removed patients was 1 (95% CrI 1–1) at day 100 and maximum number of symptomatic infectious patients per day was 0.2 (95% CrI 0–0.3) (Figure 2). R_0_ was 0.021 (IQR 0.016–0.025).

**Figure 3:**
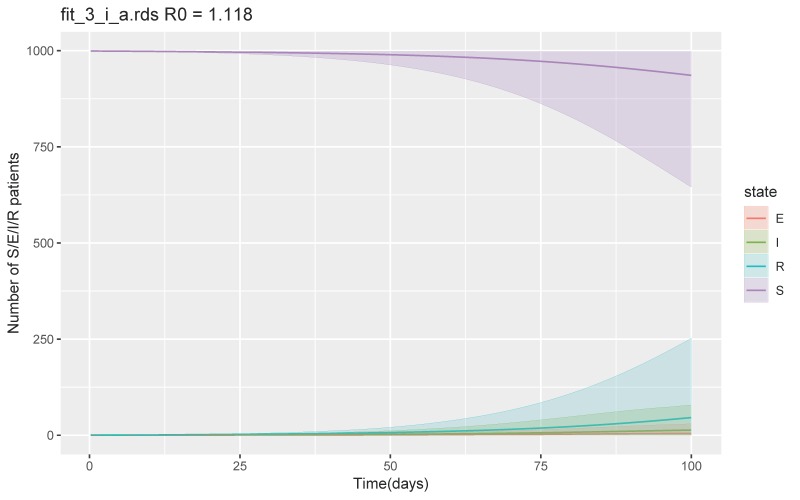
SEIR model with the parameters (β 0.1–0.2, δ 1/4–1/2, and ν 1/10–1/7) when the secondary outbreak started to occur. Abbreviations S = susceptible, E = exposed, I = infected, and R = recovered and removed.

**Figure 4:**
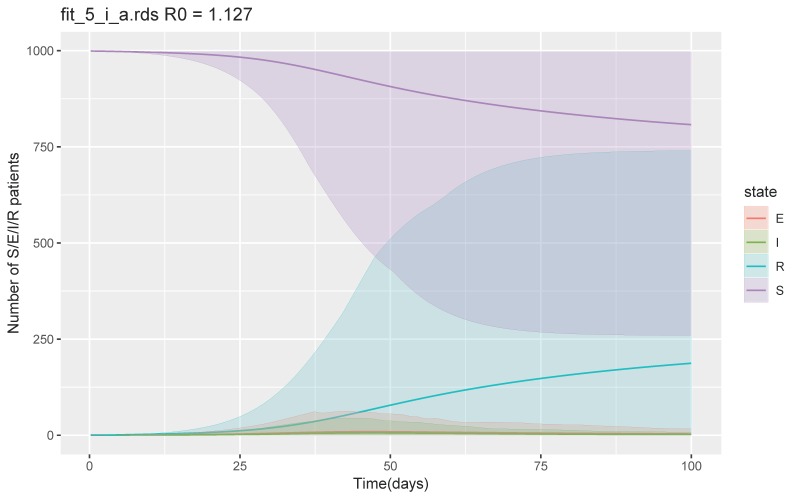
SEIR model with β of 0.8–1.0 but with δ of 1/4–1/2, and ν of 1/2–1. Abbreviations S = susceptible, E = exposed, I = infected, and R = recovered and removed.

The secondary outbreak did not occur while incrementing each parameter until the scenario arrived at β of 0.1–0.2, δ of 1/4–1/2, and ν of 1/10–1/7, when the total number of the recovered or removed patients was 46 (95% CrI 2–252) at day 100 and maximum number of symptomatic infectious patients per day was 13 (95% CrI 1–79) (Figure 3). R_0_ was 1.1 (IQR 0.8–1.4). However, when we increased β to 0.4–0.6, while keeping δ and ν the same, the secondary outbreak did not occur. With the same β of 0.4-0.6, increasing the duration until medical visit from 2 to 7 days, and 7 to 10 days (ν 1/7–1/2, 1/10–1/7 respectively), the total number of recovered or removed patients increased to 304 (95% CrI 2–919) and 874 (95% CrI 247–994) respectively, with R_0_ of 1.4 (IQR 0.9–1.7) and 3.4 (IQH3.4 (IQR 2.5–4.2) respectively. Increasing β to 0.8–1.0 with δ of 1/4–1/2, and ν of 1/2–1 led to a total of 187 patients recovered or removed at day 100 (CrI 3–740) with R_0_ of 1.1 (IQR 0.9–1.3) (Figure 4). The rest of the simulations and calculated R_0_ are shown in the supplementary file.

## 4. Discussion

The number of new patients diagnosed with 2019-nCoV infection keeps increasing as of this writing [1]. Several studies estimated basic reproductive number (R_0_) by different methods and they range from 2.2 to 5.5 [9,11,12,13,14,15,16,17] (Table 1). However, the R_0_ in countries outside China has not been fully investigated as of this writing [2,3,4,5,6,7,8]. Since R_0_ is determined not only by virologic nature and biological characteristics of hosts, but also by other factors, such as the number of close contacts with the infected, use of protection such as surgical masks, or timely isolation of patients at health care facilities, published mathematical models based on existing coronavirus and other epidemics within China might not apply to 2019-nCoV infections in a different setting. Our simulation with multiple scenarios indeed demonstrated that shortening the duration from the onset of symptoms to medical visit could decrease the number of infected even with the same level of force of infection. For example, our simulations suggested if β was 0.8–1, and the patient spent 7 to 10 days without attending medical services, almost all in the community of 1000 would be infected within 2 months. If β was the same, but a patient spent only 2 to 4 days before visiting a hospital, only less than 200 might be infected by day 100. With advanced reporting systems and adequate infection control practices in health care facilities, transmissibility of this virus could be decreased in other developed countries compared to Wuhan. Therefore, the estimation of the risk of secondary outbreaks separately, as we have done, has value in infection control in other nations.

Our results suggest that hundreds of newly diagnosed 2019-nCoV infections could occur within a bad scenario when a person with the infection enters into a community of a thousand. This means we need to be alert to the possibility that secondary transmission and spread of this virus is a very realistic possibility and be prepared for this future possibility. On the other hand, we also demonstrated that, with a good scenario, a secondary outbreak might not occur, as seen in most countries which have had 2019-nCoV imported cases as of this writing. Improving the alert system with early recognition of the infected persons could decrease or hold a secondary outbreak.

Our study does have several inherent limitations. First, because most parameters regarding 2019-nCoV remain unknown, especially those in different countries other than China, we had to rely on arbitrary hypothetical numbers, and they may not be accurate. A table compares our scenarios with estimated/assumed parameters used in studies for patients in China (Table 1). Second, there is a hypothesis that asymptomatic patients could be infectious to others [18], although how frequently this occurs remains unknown. We did not incorporate this hypothesis into our analysis. Third, we assumed that there would be no nosocomial outbreak of 2019-nCoV in the region we hypothesized, presuming adequate infection control practices could prevent this occurring. However, breach of infection control practice could happen in any area and we have had nosocomial outbreaks of imported infections in developed nations such as Canada [19], the United States [20], and South Korea [21]. Fourth, we hypothesized that 2019-nCoV infection would lead to fairly long-lasting immunity and did not assume the possibility of re-infection to the infected persons. With these limitations, we need to gain more data, and to study the risk of this new infection and the virus further.

## 5. Conclusions

Hundreds of secondary infections by 2019-nCoV are possible in a developed country when a person with the infection is introduced into a community. Adequate reporting systems and infection control practice, however, could mitigate the risk to a certain level according to our simulations.

## Figures and Tables

**Table 1:** Published estimation of basic reproductive numbers and available parameters of 2019-nCov and their settings (as of 16 February 2020).

Author	β	δ	ν	Estimated Basic Reproduction Number (R_0_)	Settings
Tang et al. [9]	1/7	N/A	0.13	6.47 (95% CI 5.71–7.23)	Mainland China
Liu et al. [11]	N/A	1/4.8	1/2.9	2.90 (95%CI: 2.32–3.63) and 2.92 (95%CI: 2.28–3.67) for different estimations.	China
Li et al. [12]	N/A	1/5.2	1/12.5 and 1/9.1 for different periods	2.2 (95% CI, 1.4 to 3.9).	Wuhan, China
Wu et al. [13]	N/A	1/6	1/2.4	2·68 (95% CrI 2·47–2·86)	Wuhan
Zhao et al. [14]	N/A	N/A	N/A	2.24 (95%CI: 1.96–2.55) to 3.58 (95%CI: 2.89–4.39)	Mainland China
Riou et al. [15]	N/A	N/A	N/A	2.2	China
Zhao et al. [16]	N/A	N/A	N/A	2.56 (95% CI: 2.49–2.63)	Mainland China
Zhou et al. [17]	N/A	1/5.1	N/A	2.8–3.3 and 3.2–3.9 *	China before the intervention (26 January 2020)

Parameters could be unavailable due to different methodology used for the analyses. CrI, credible interval. CI, confidence interval. N/A, not available. * Two sets of R_0_ were calculated for 2 different publications.

## Supplementary Materials

Supplementary materials can be found at https://www.mdpi.com/2077-0383/9/4/944/s1.
