# Supplementary figures and images for: A Simulation on Potential Secondary Spread of Novel Coronavirus in an Exported Country Using a Stochastic Epidemic SEIR Model
Source: J Clin Med. 2020 Mar 30;9(4):944. doi: 10.3390/jcm9040944 (PMC7230280; doi:10.3390/jcm9040944)

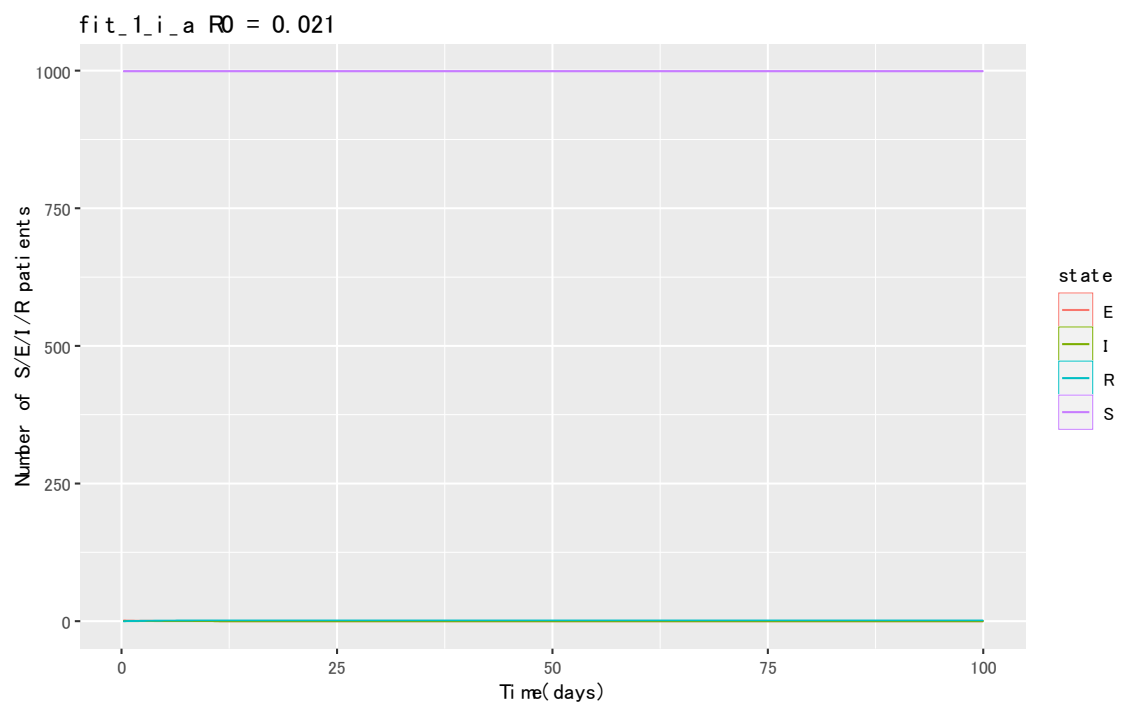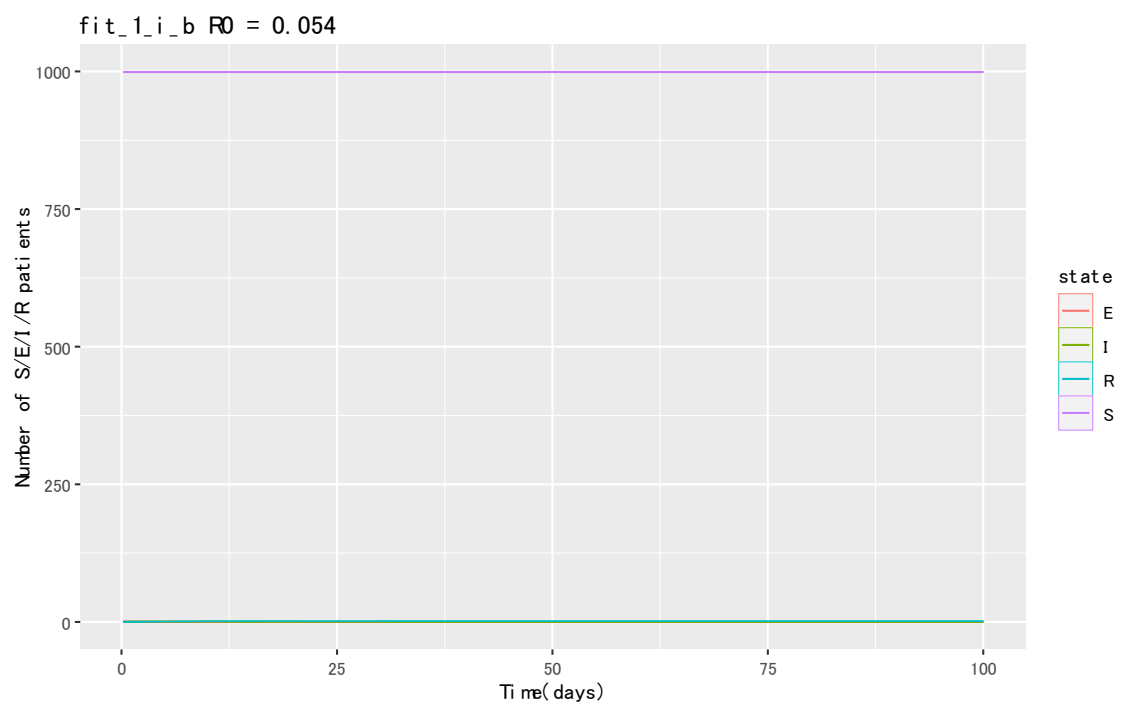

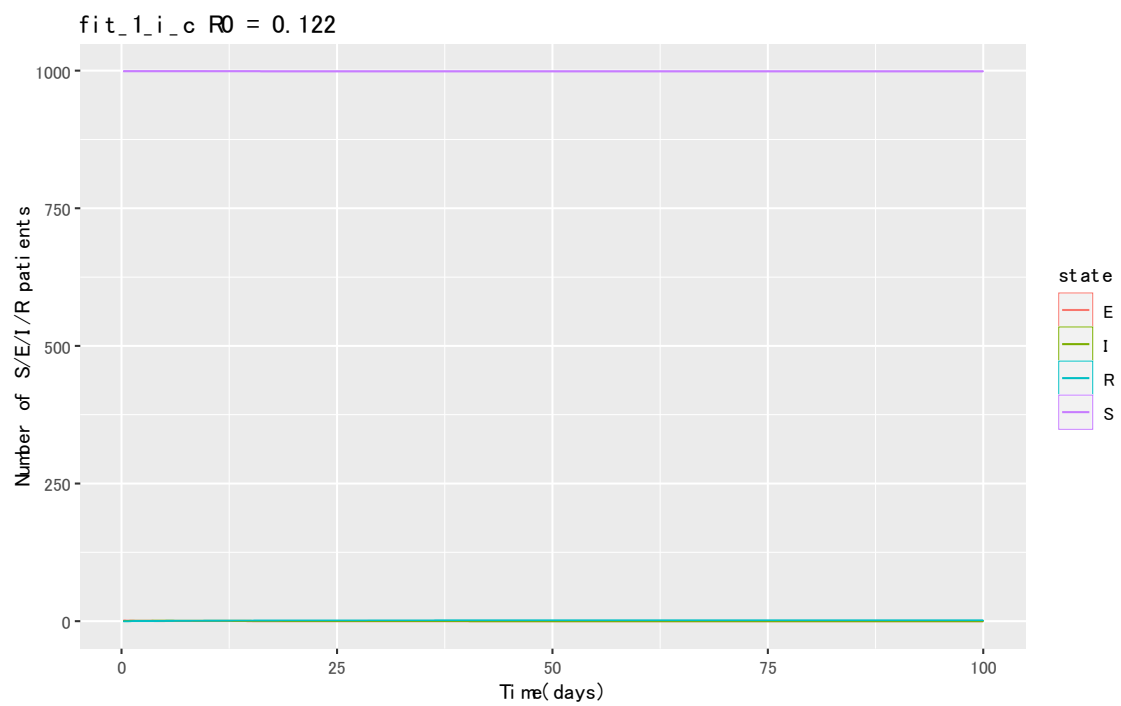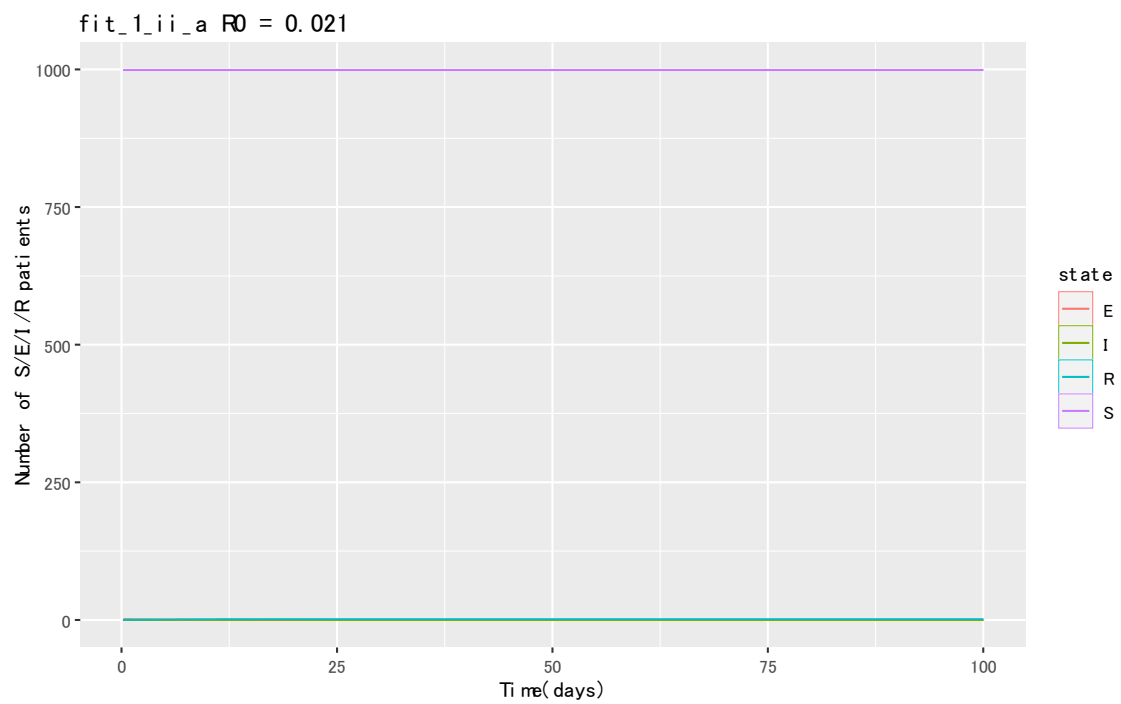

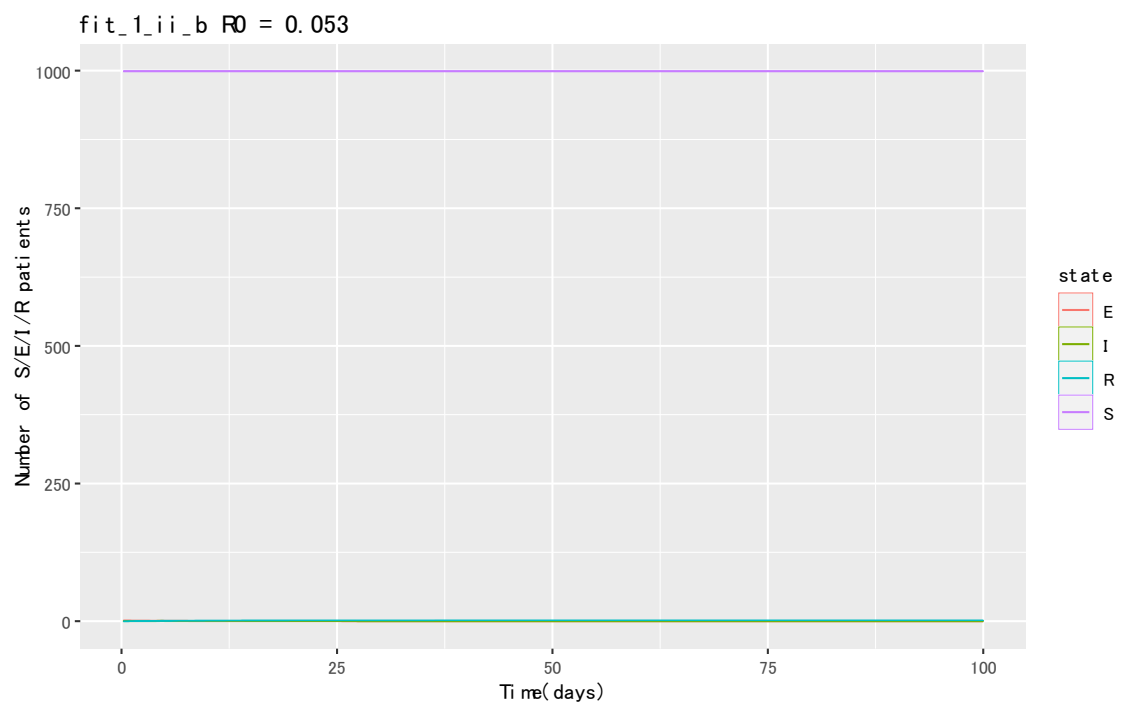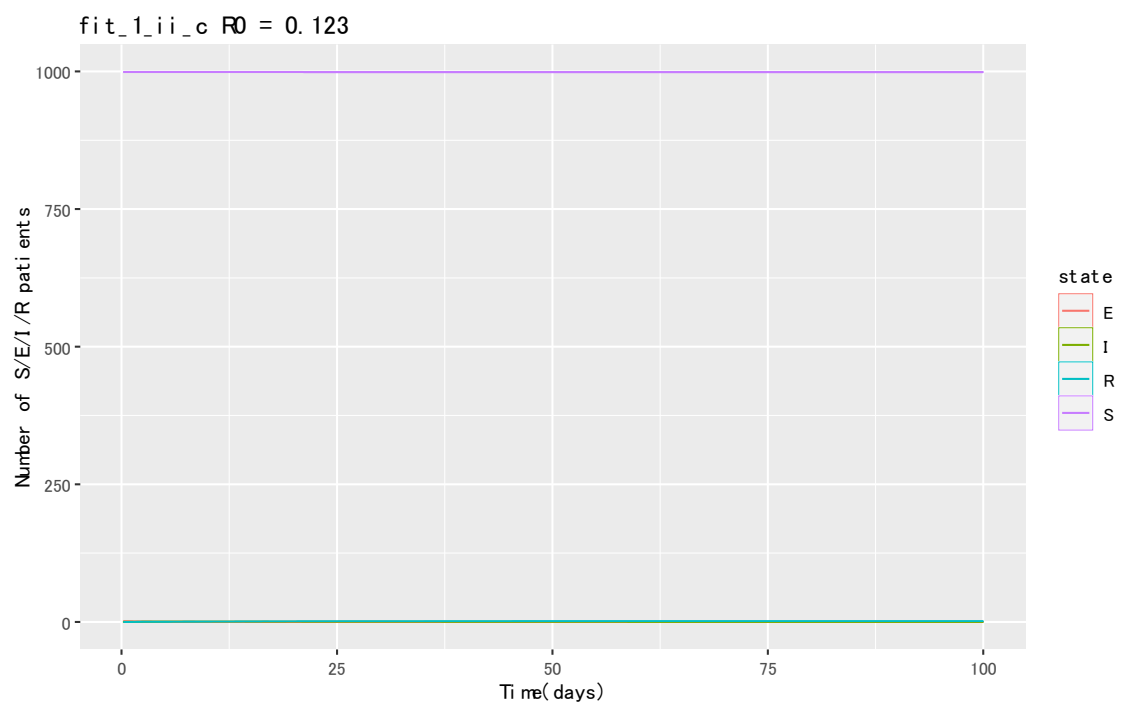

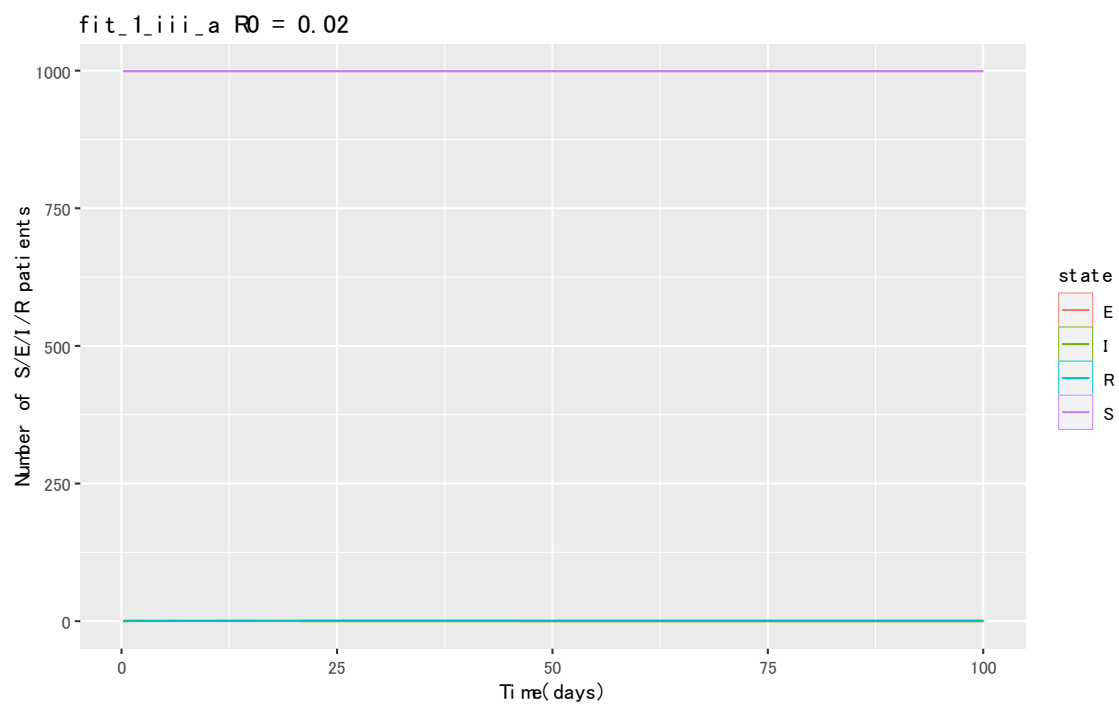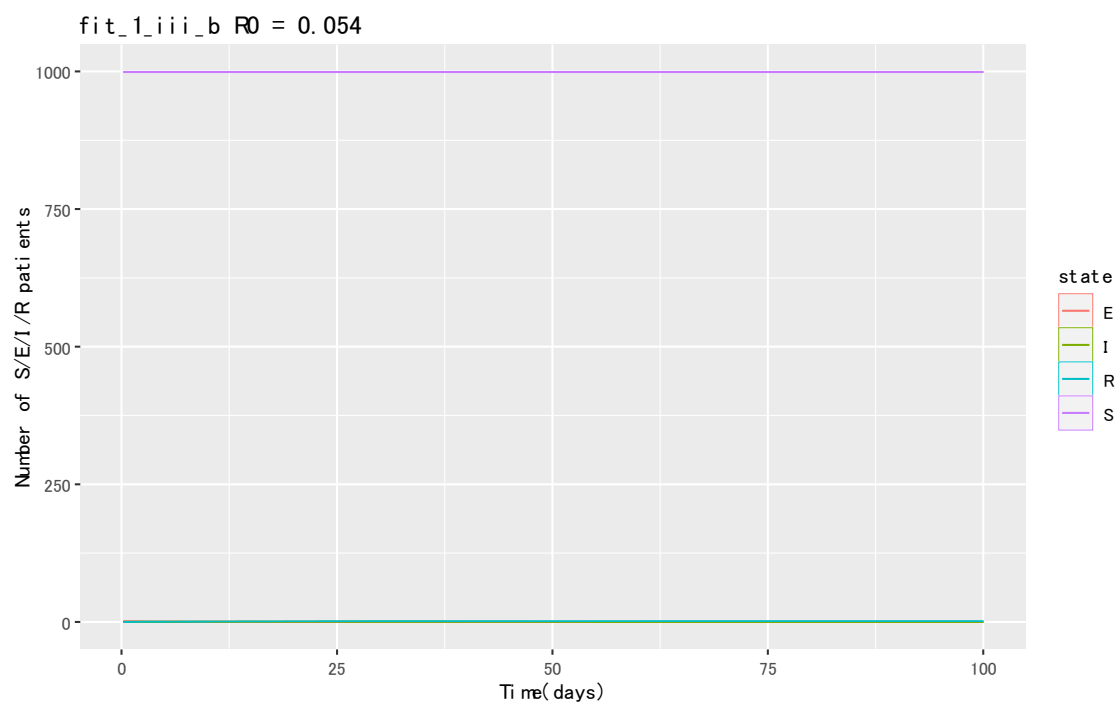

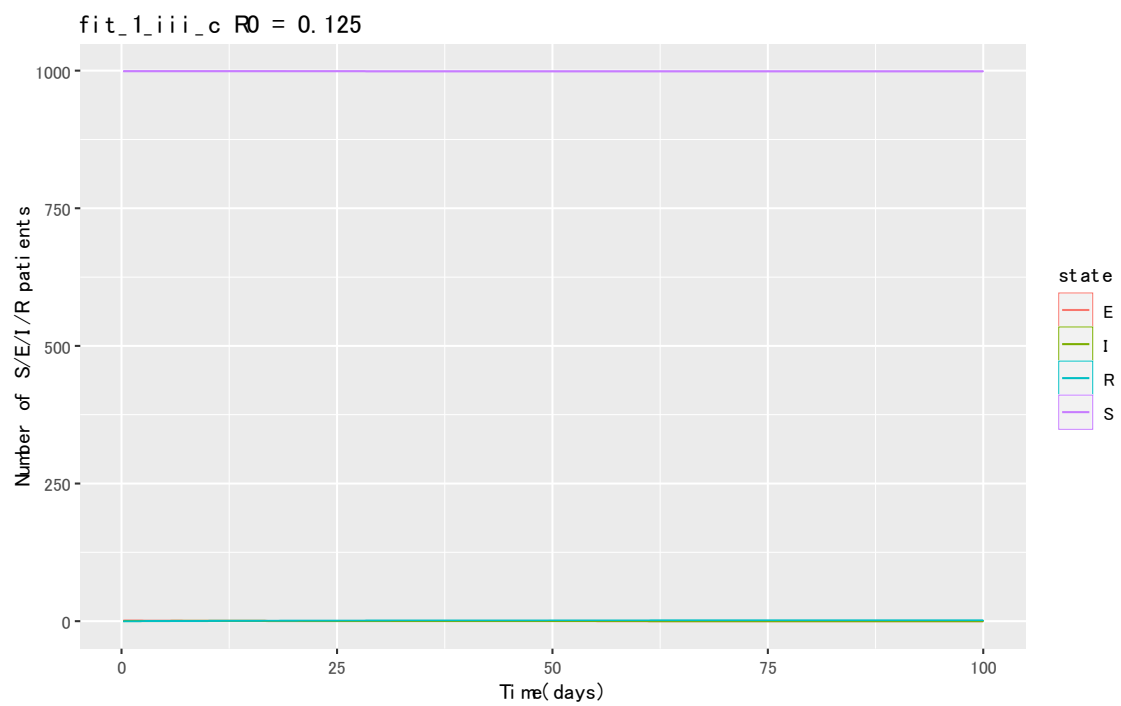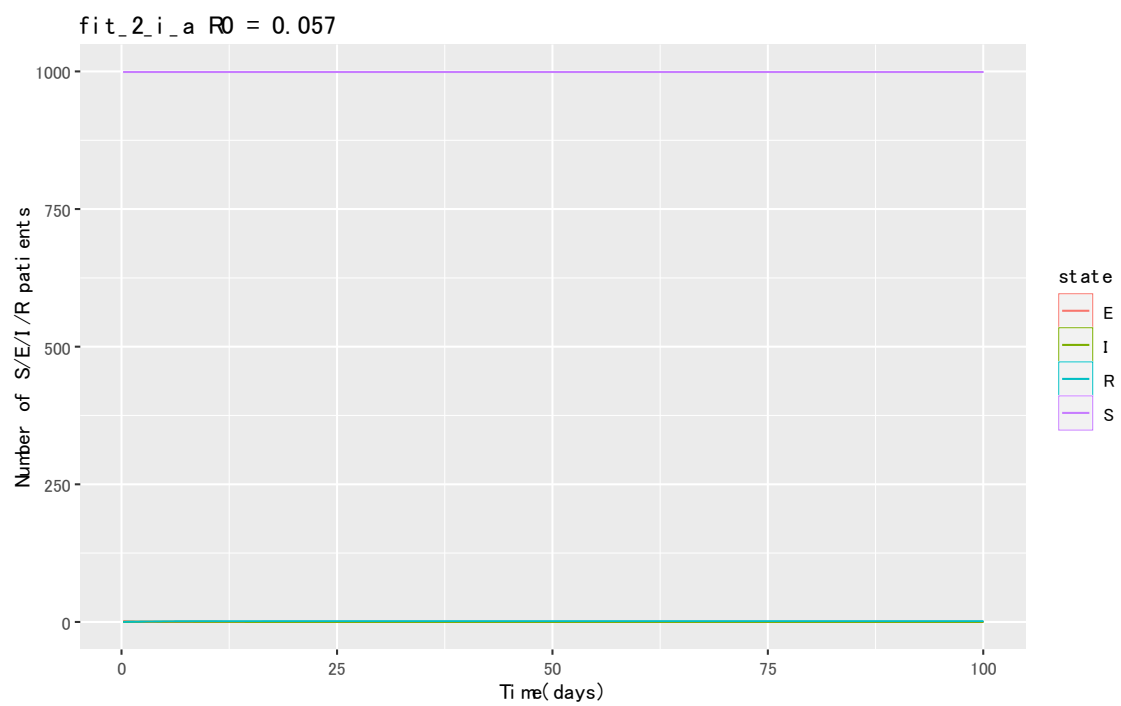

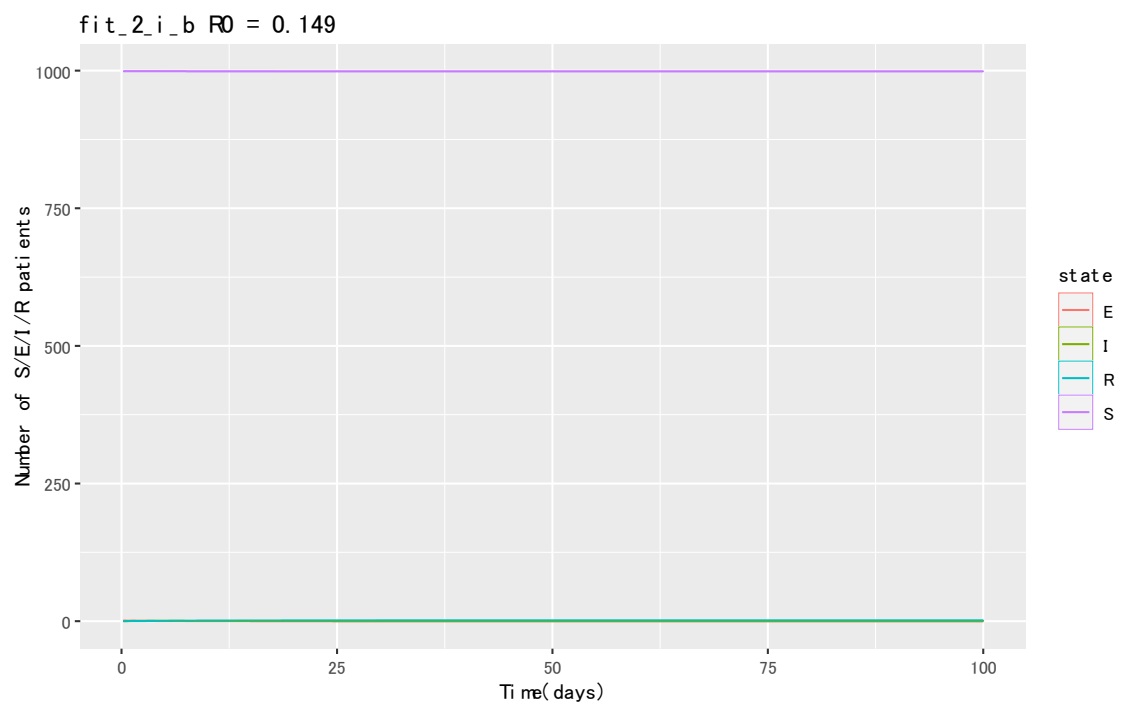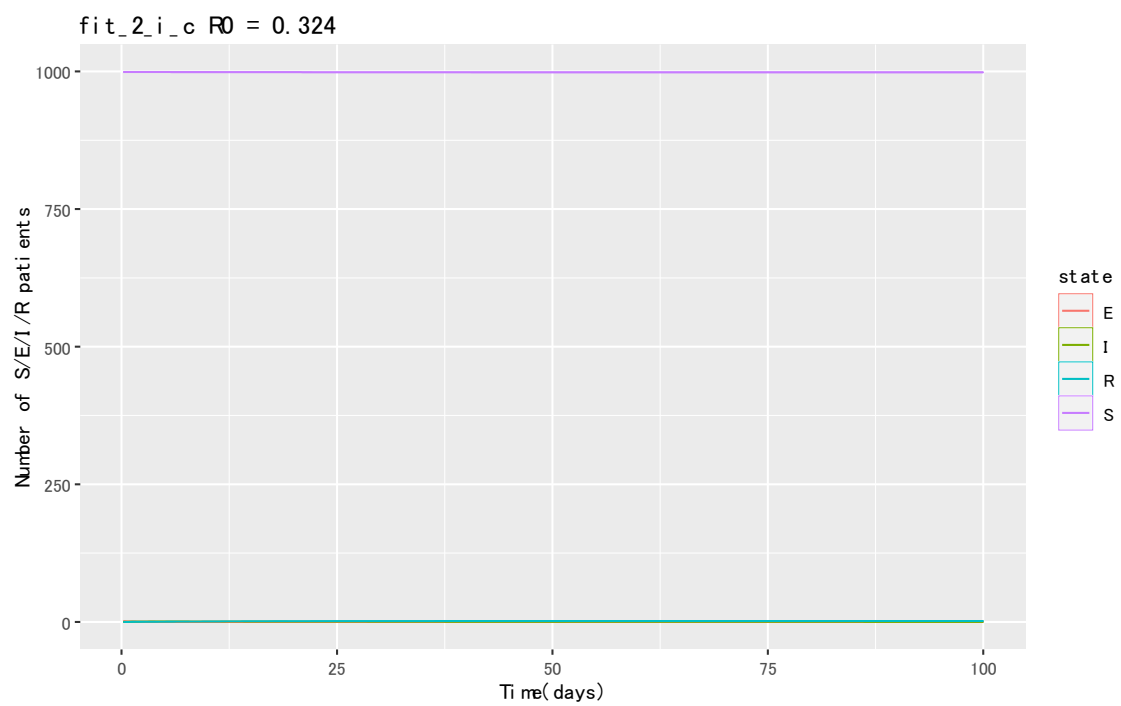

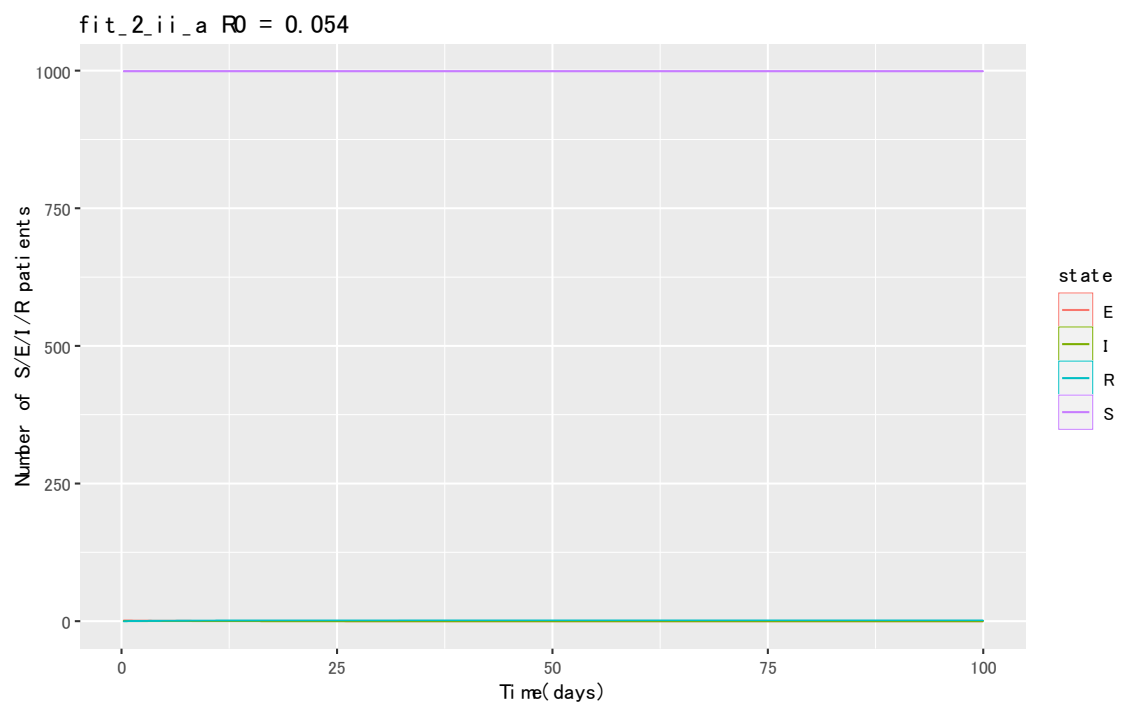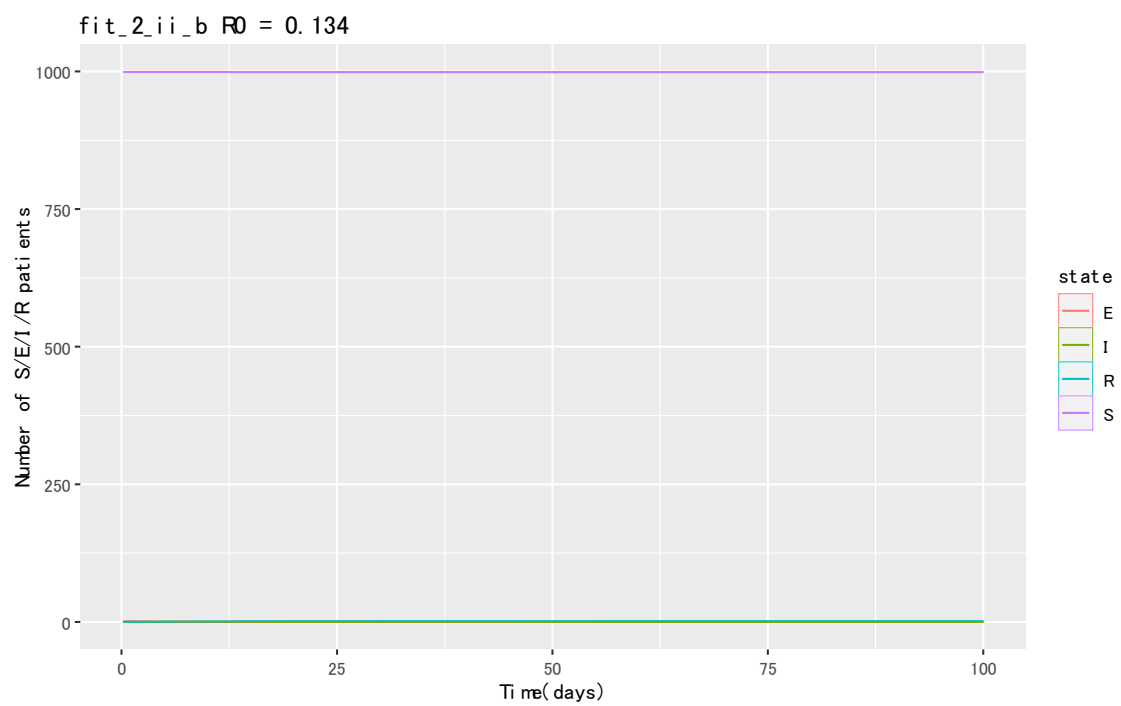

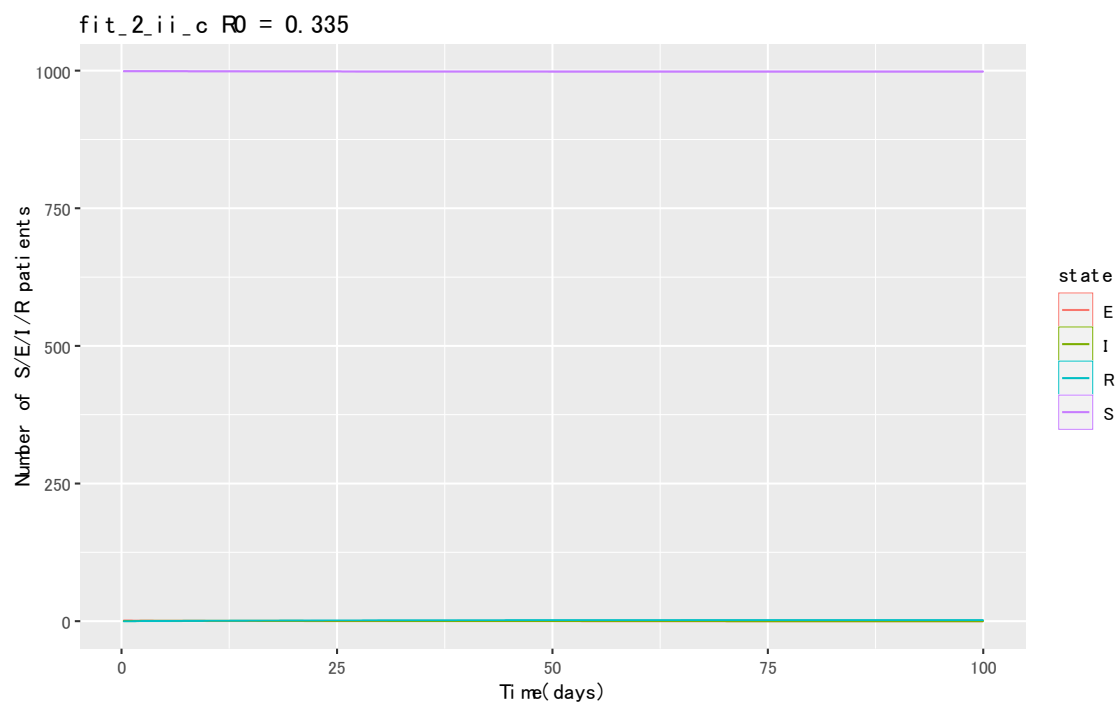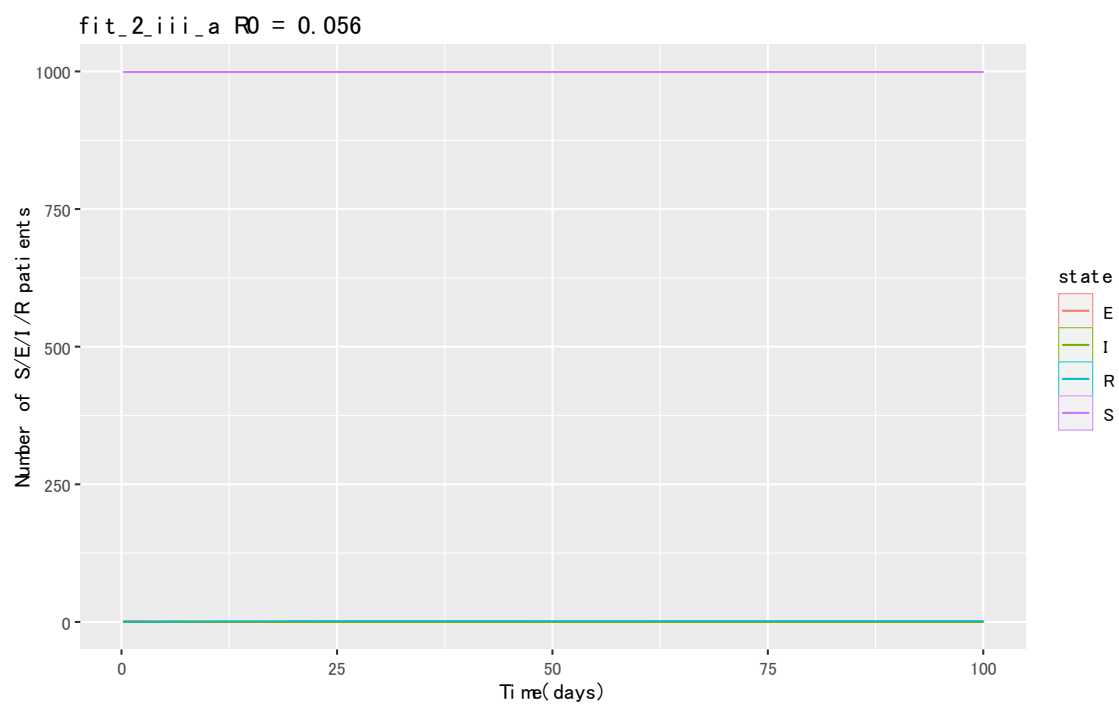

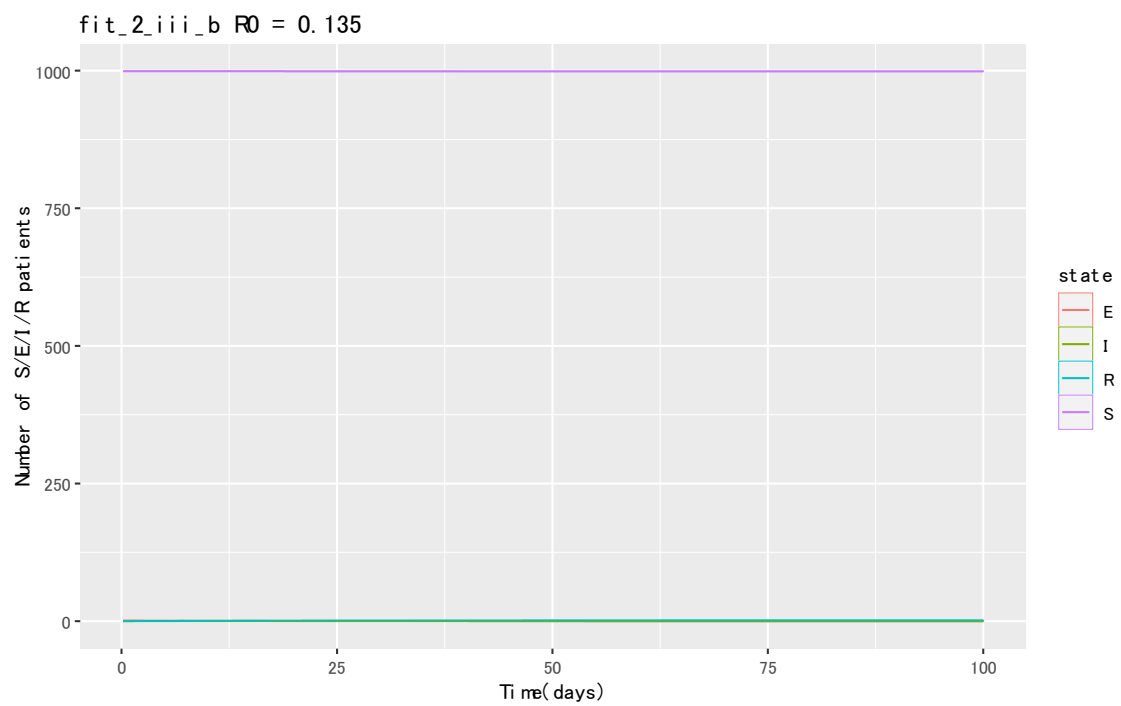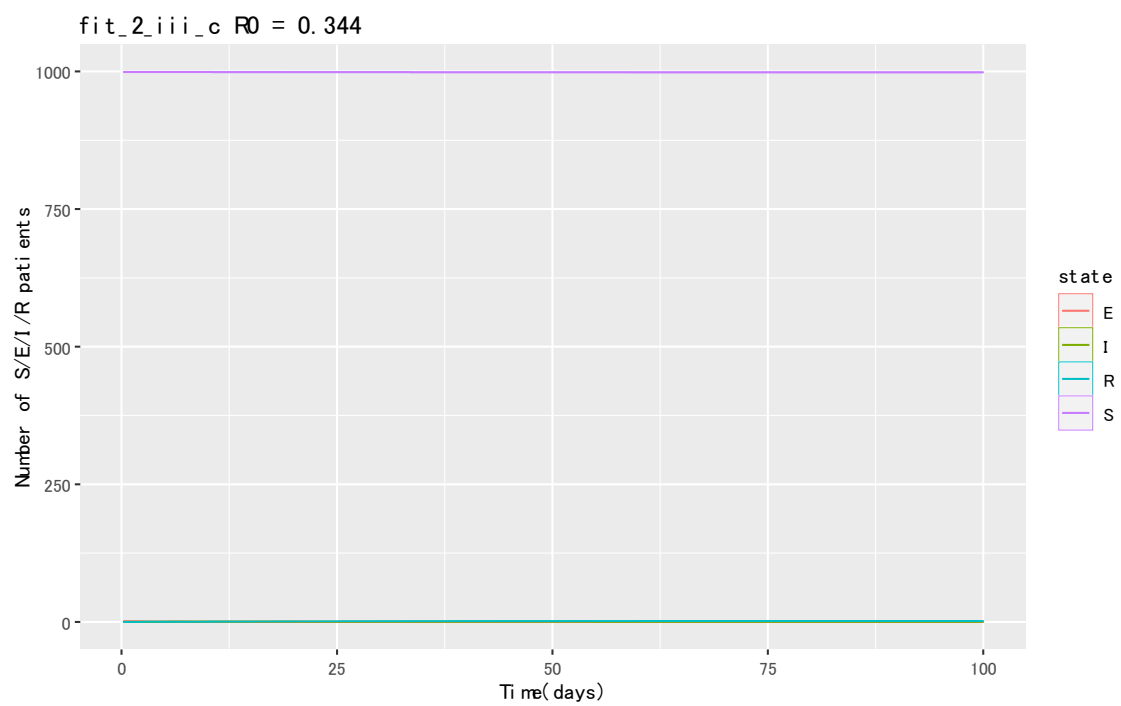

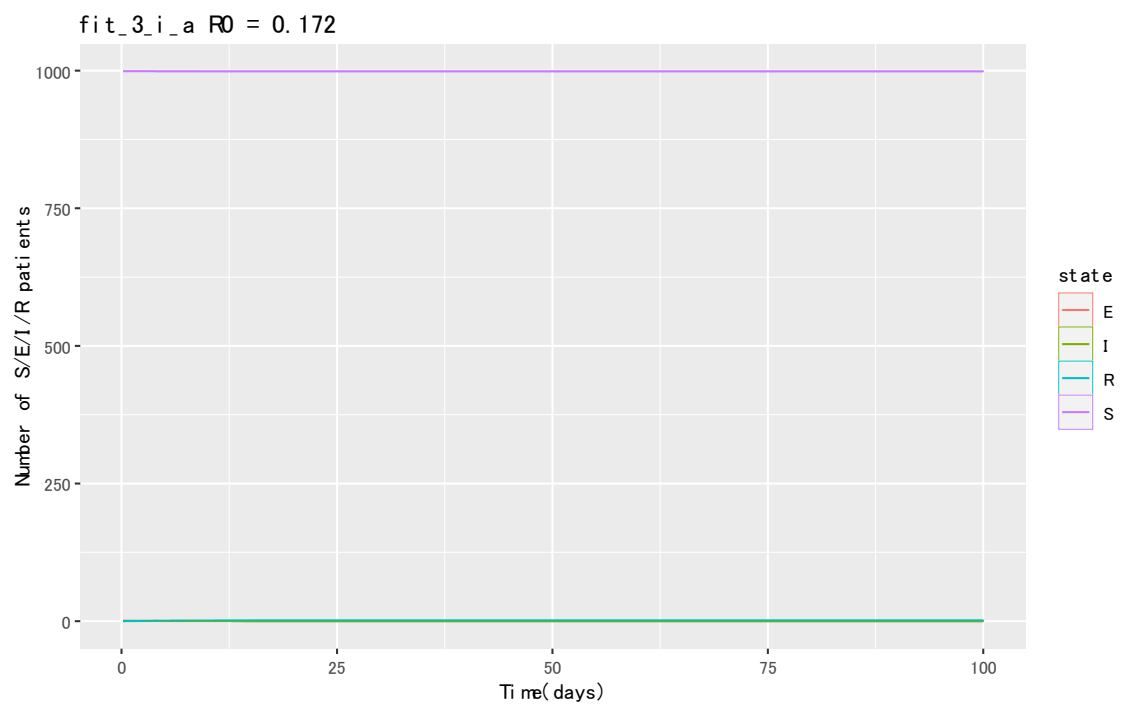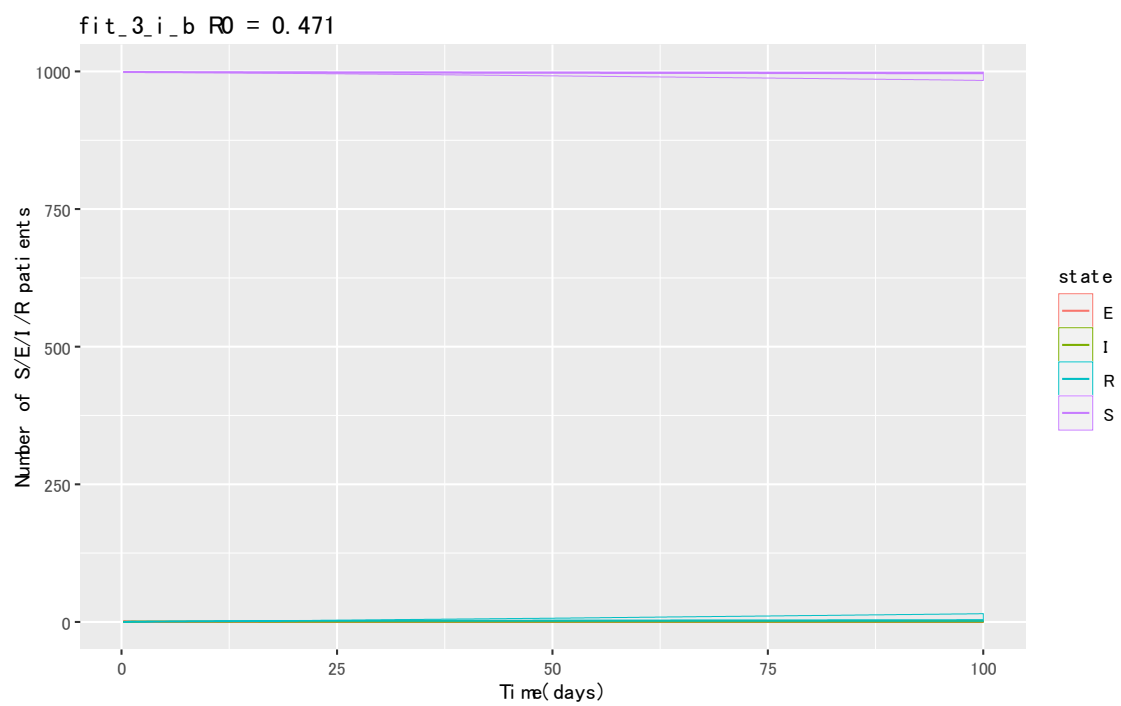

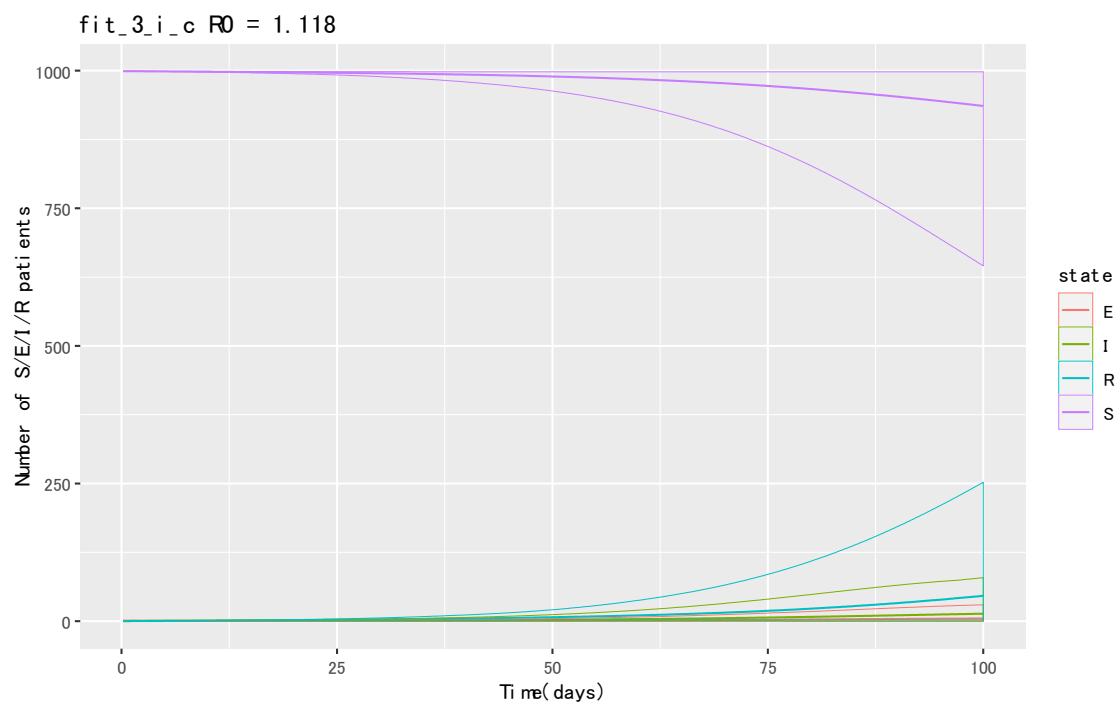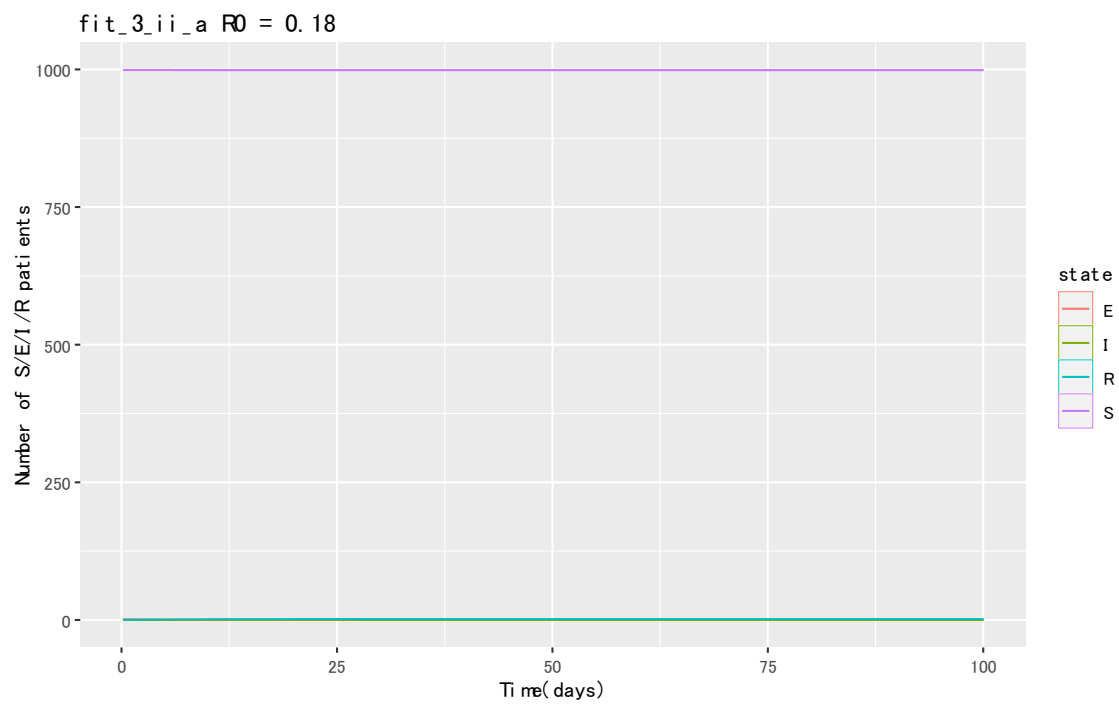

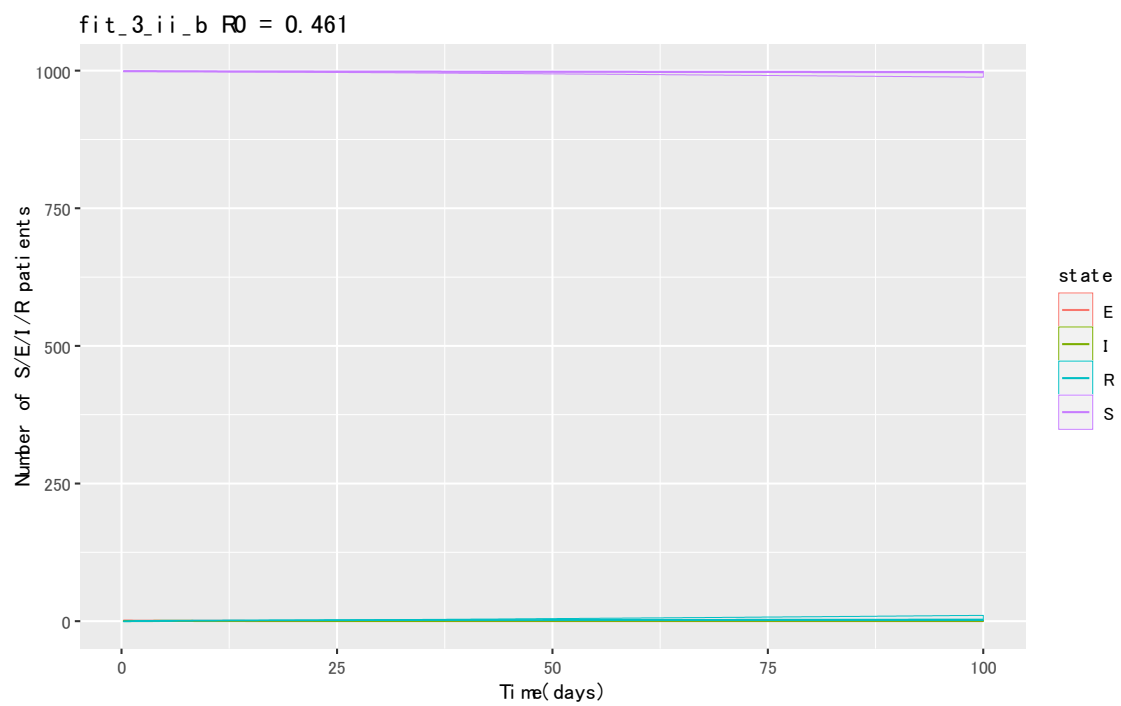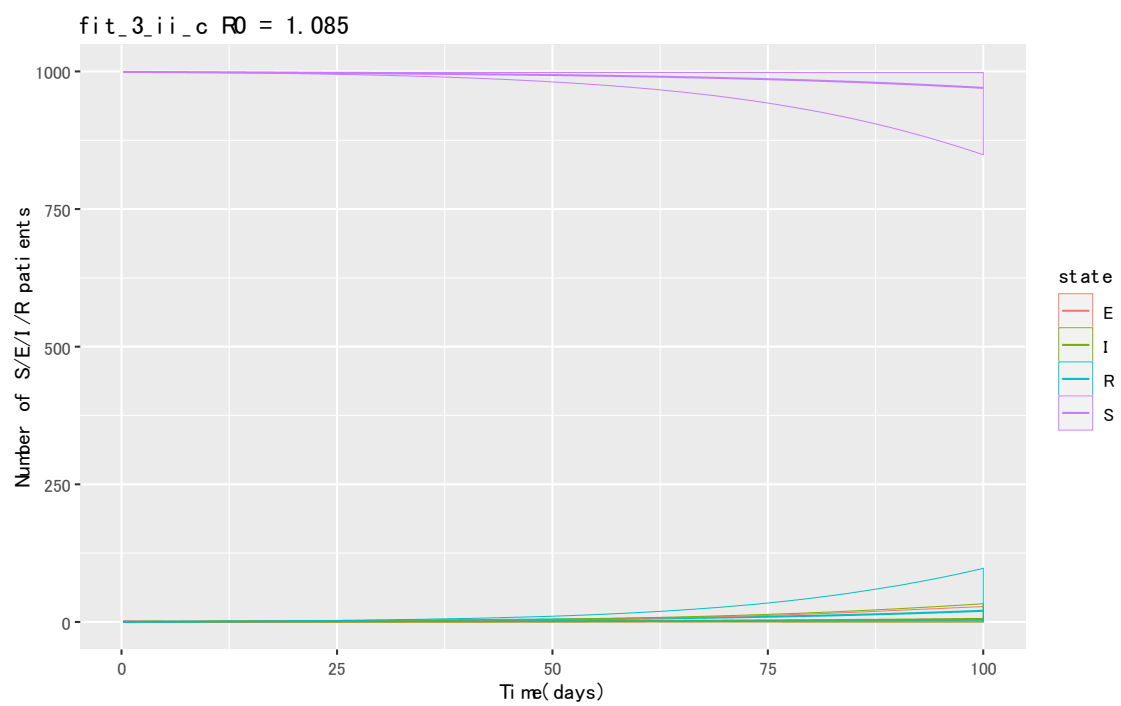

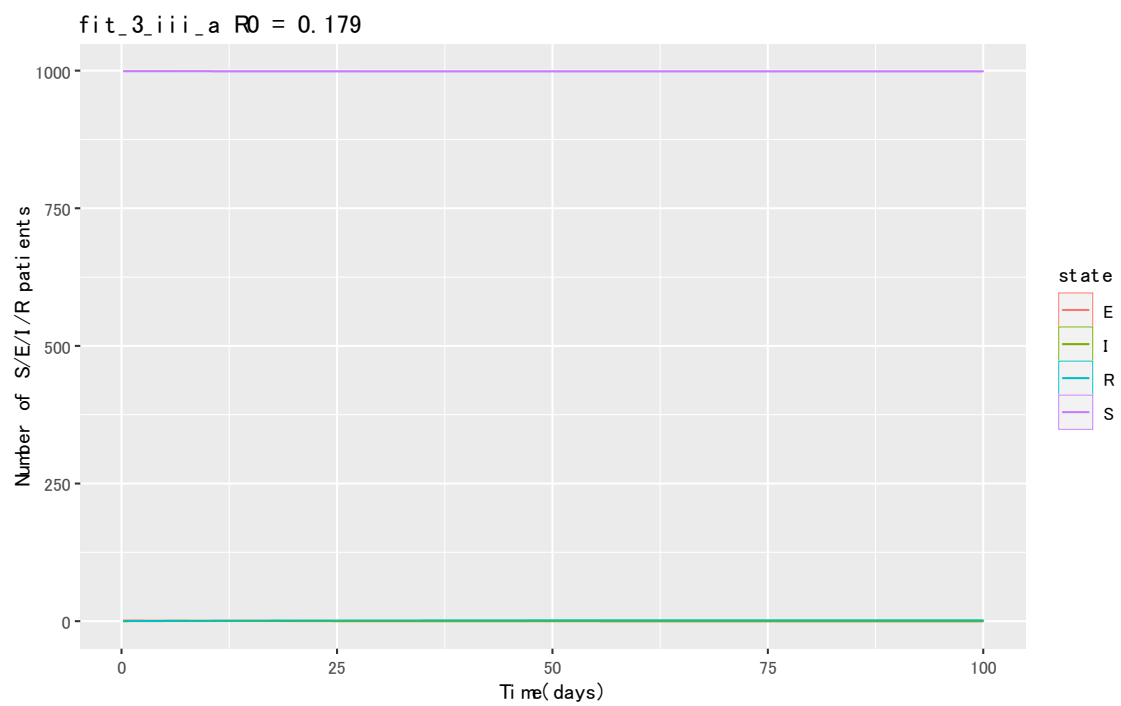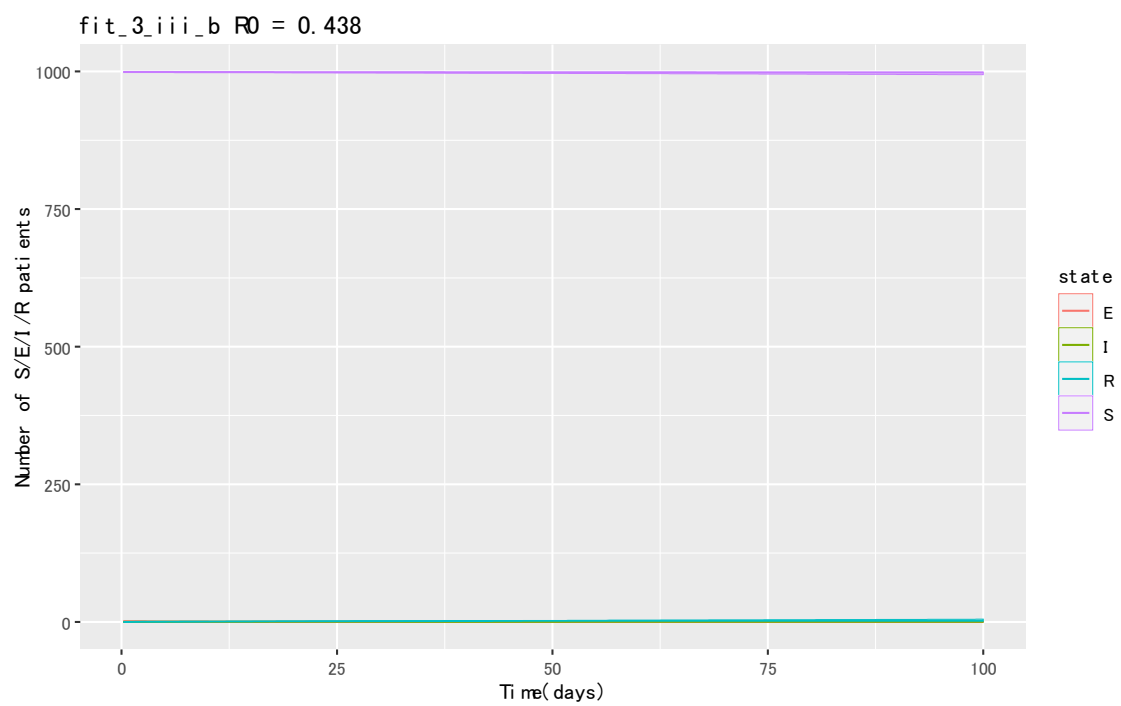

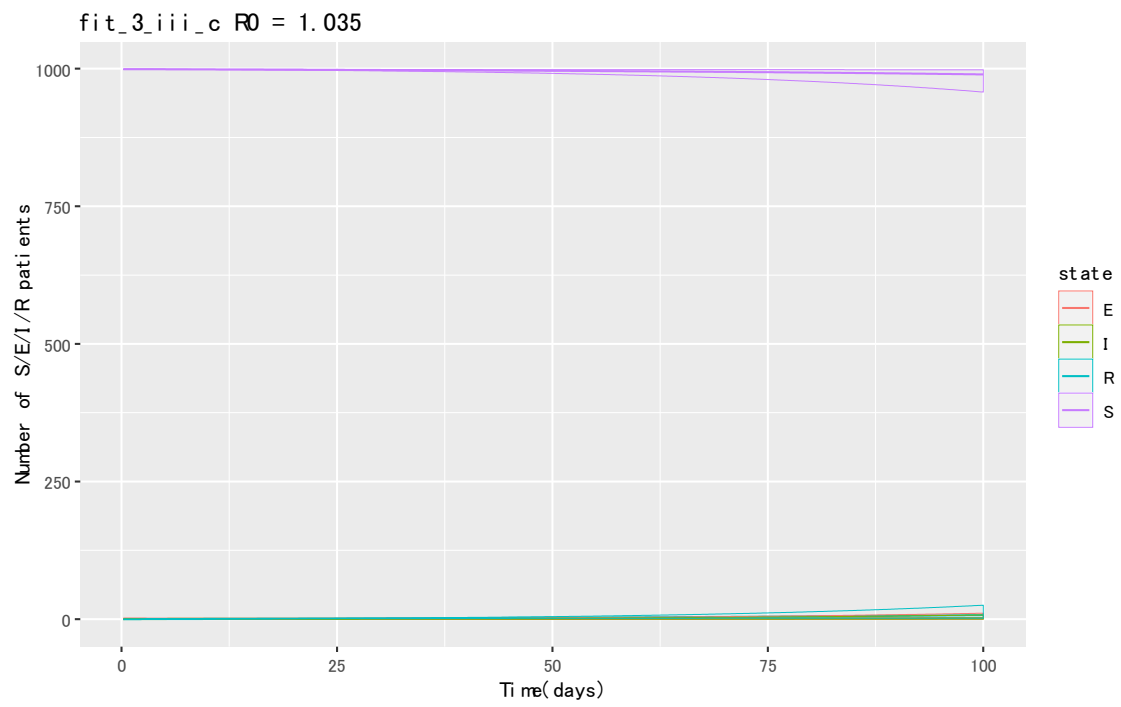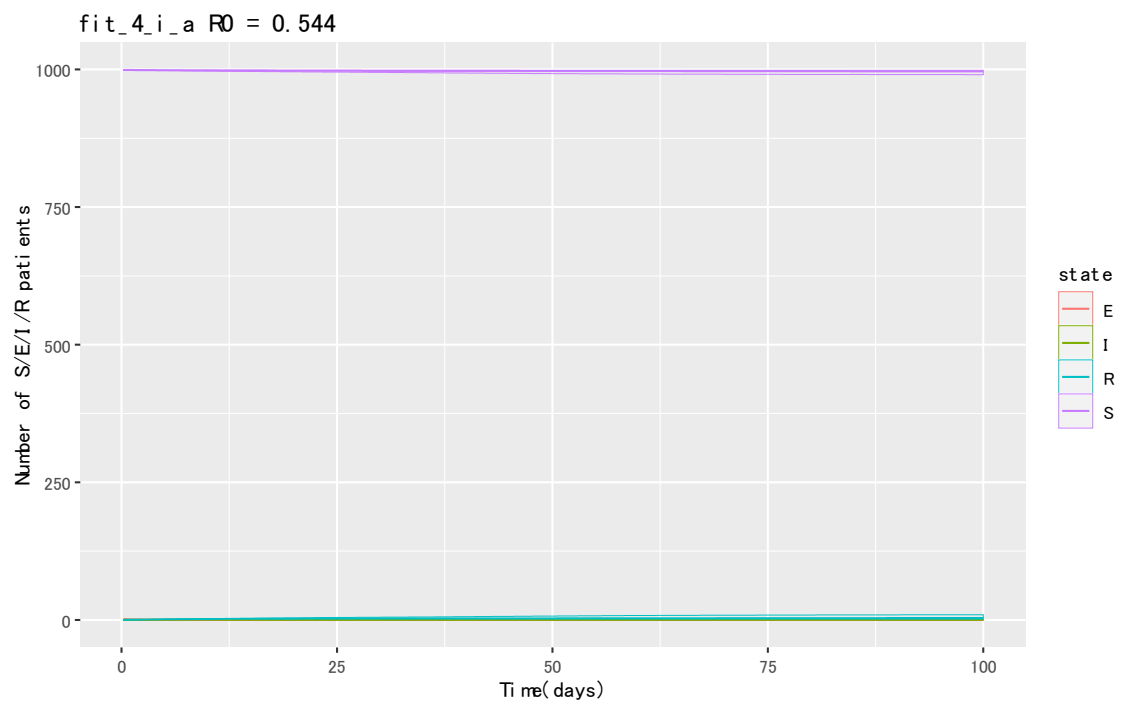

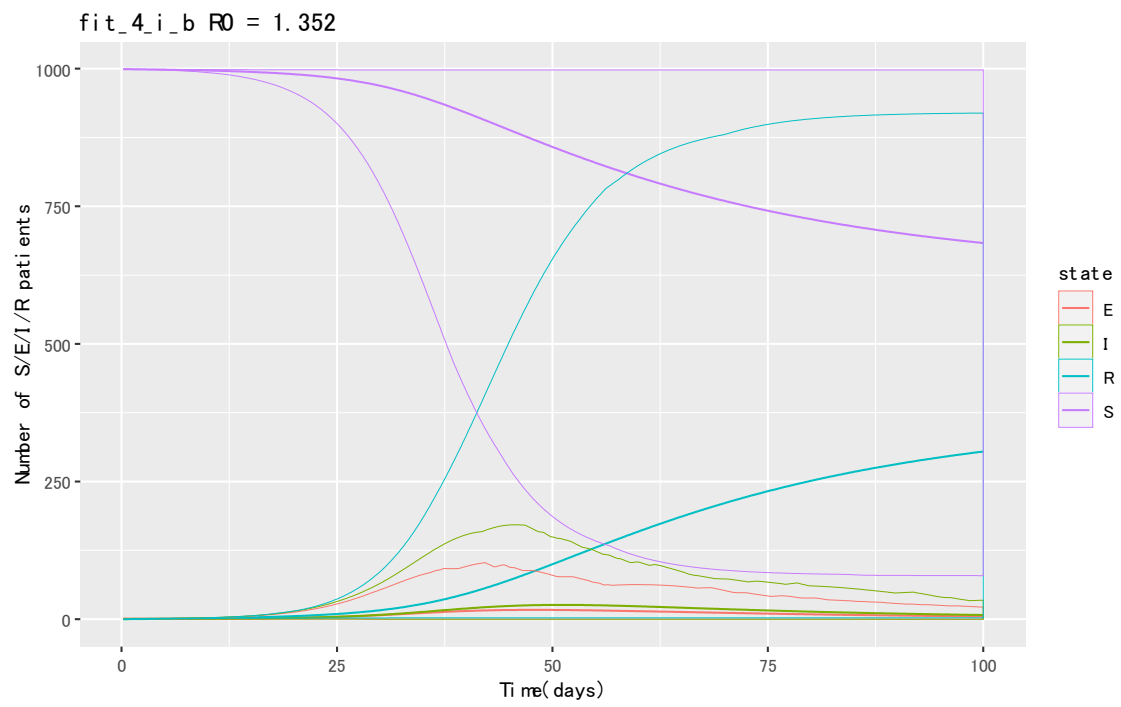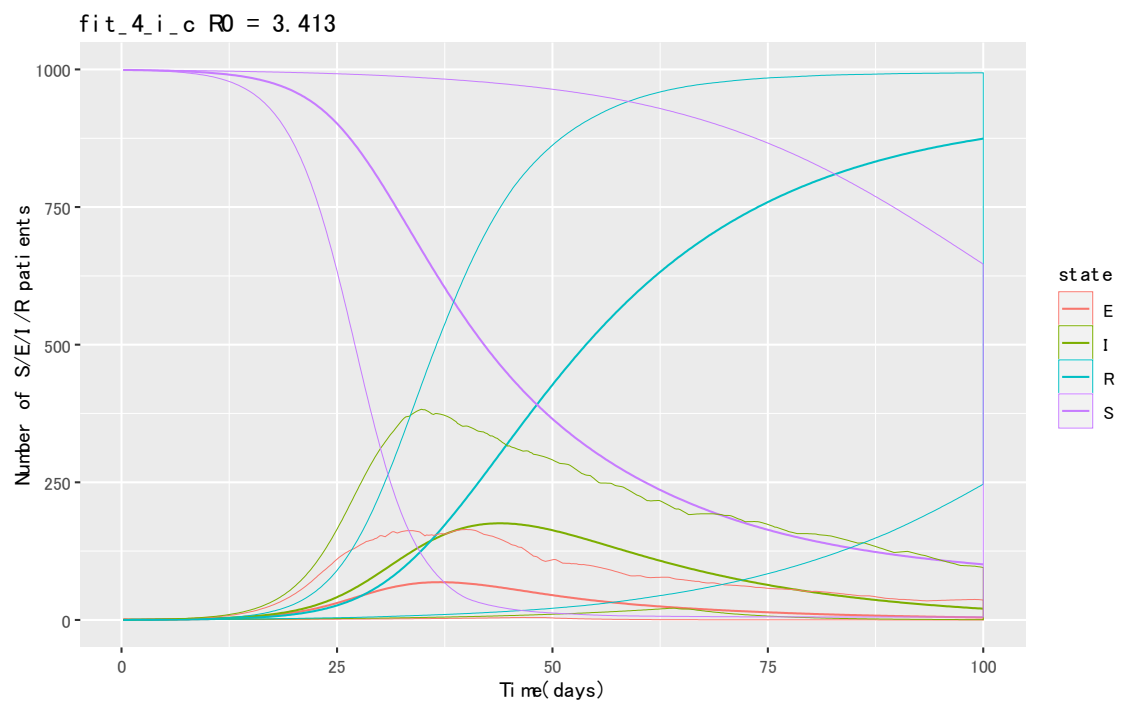

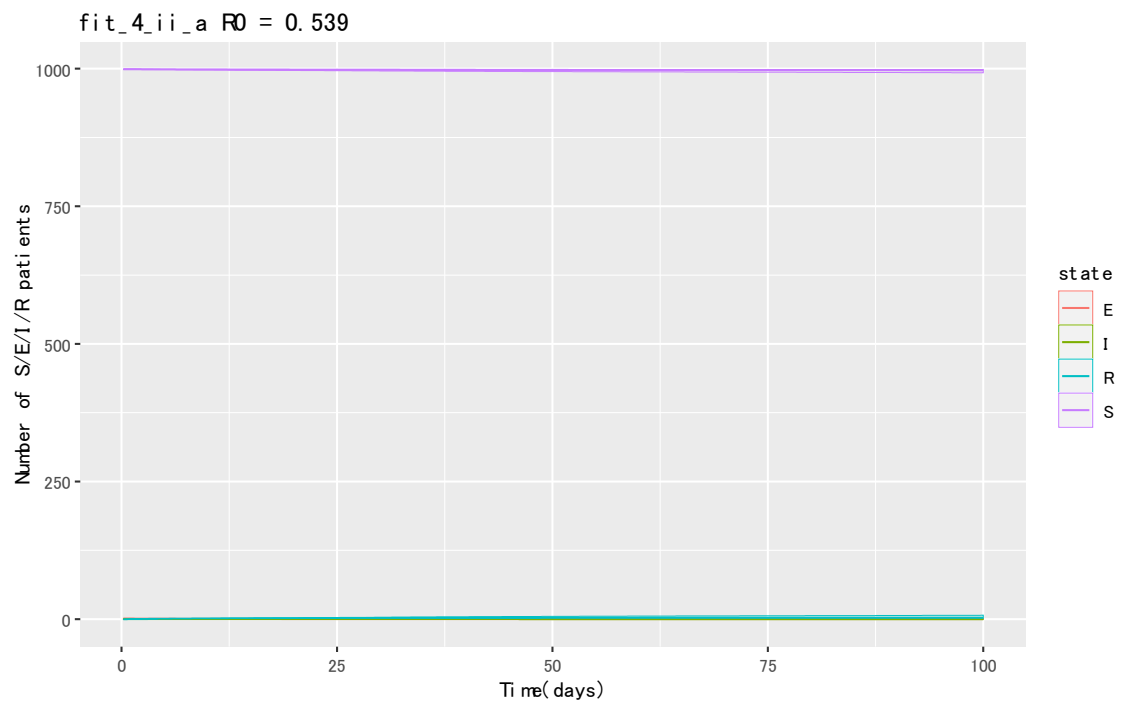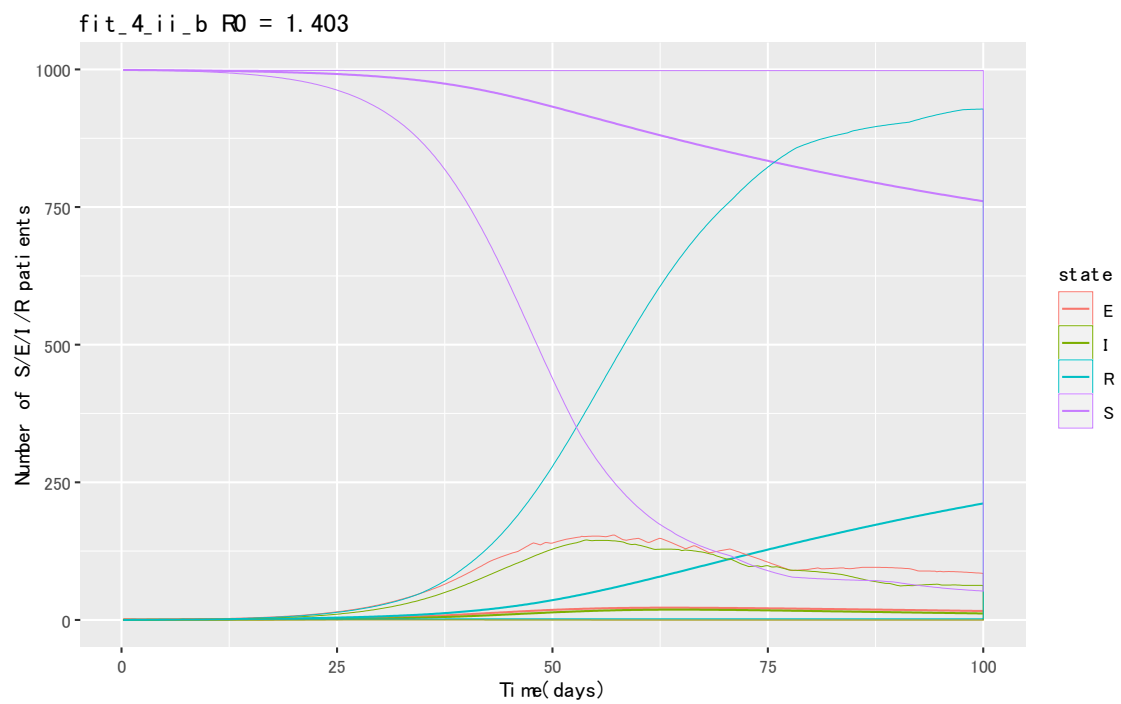

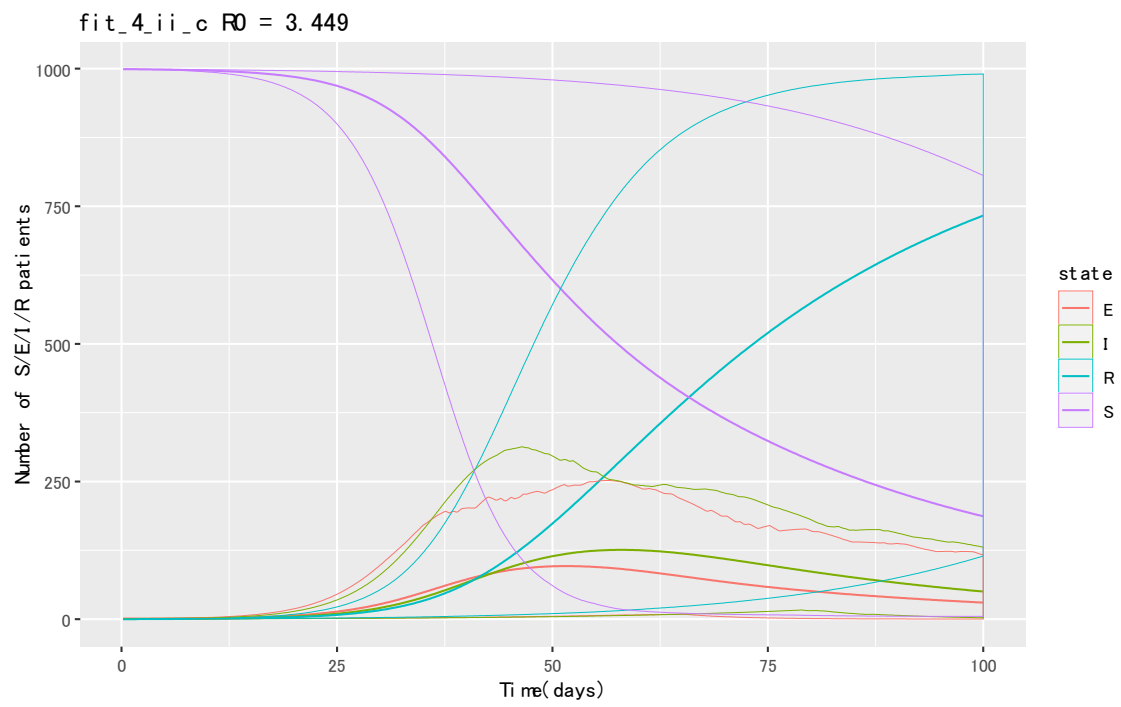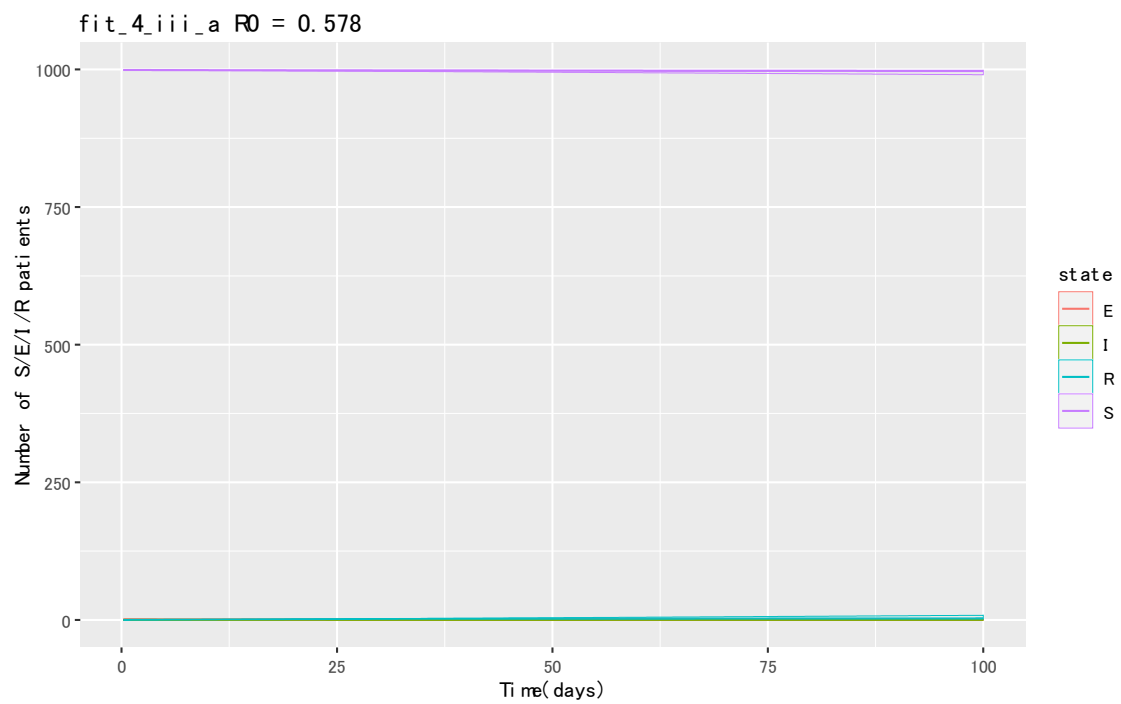

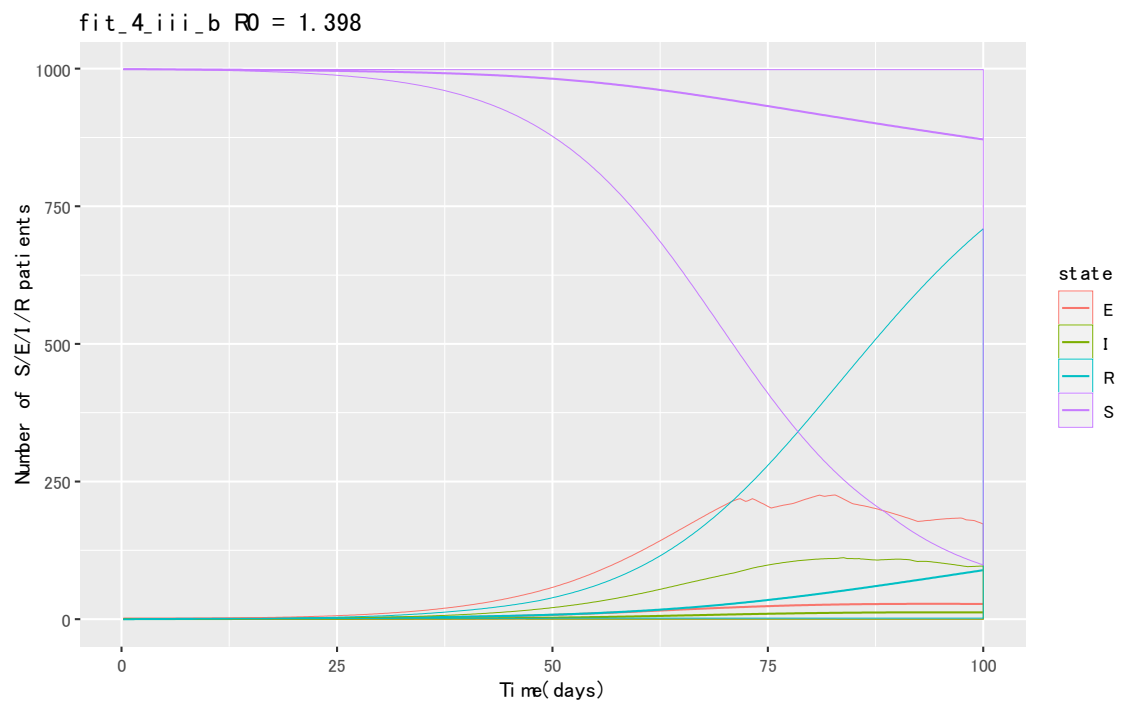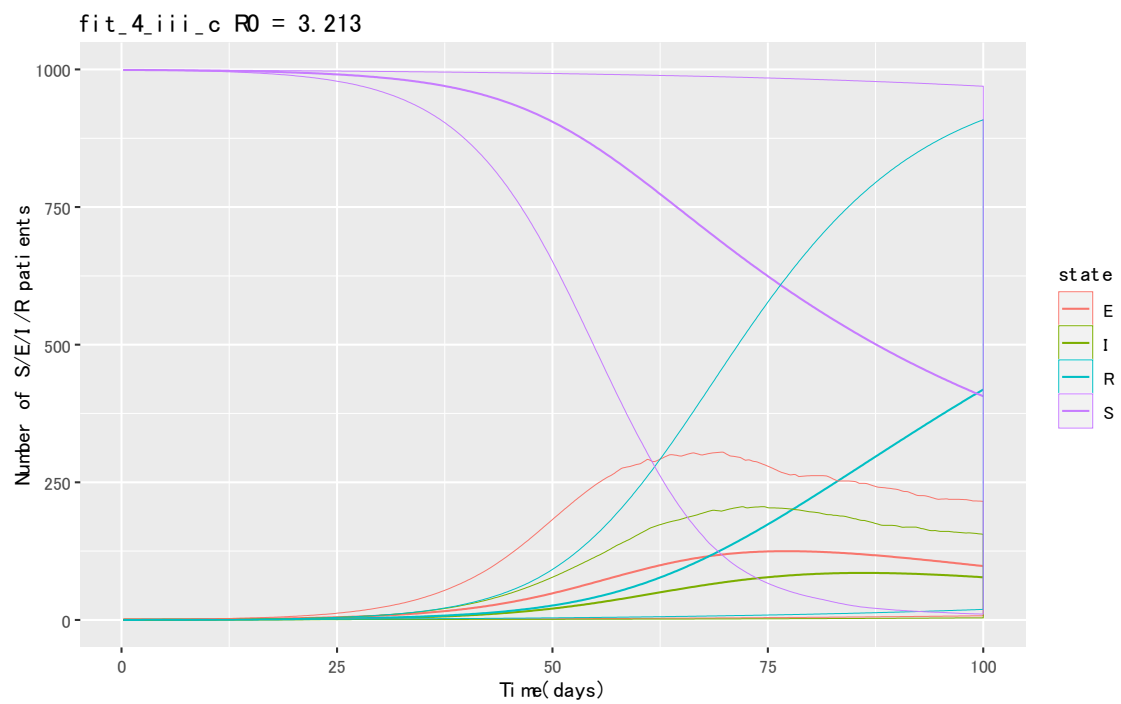

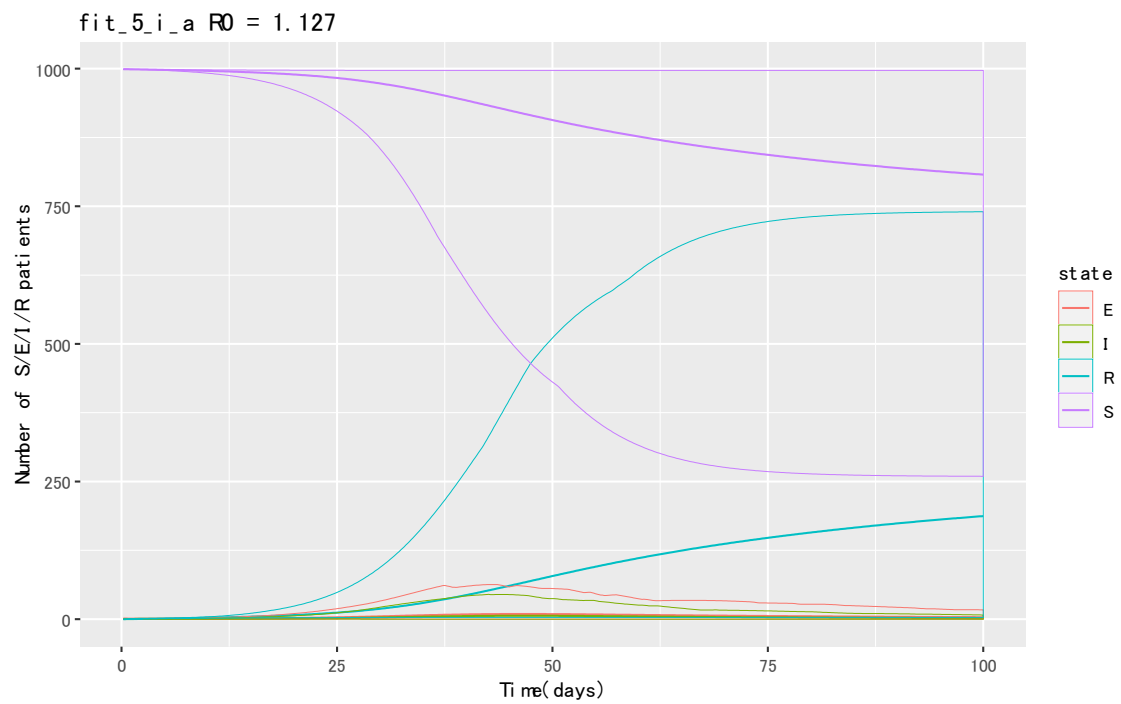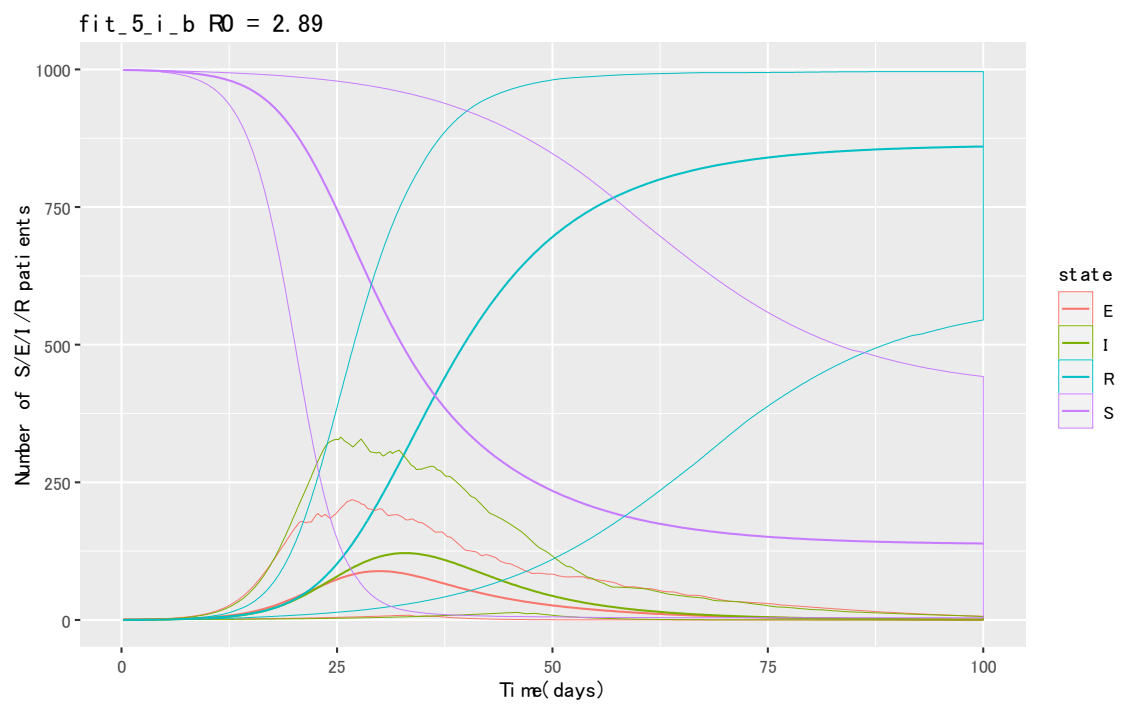

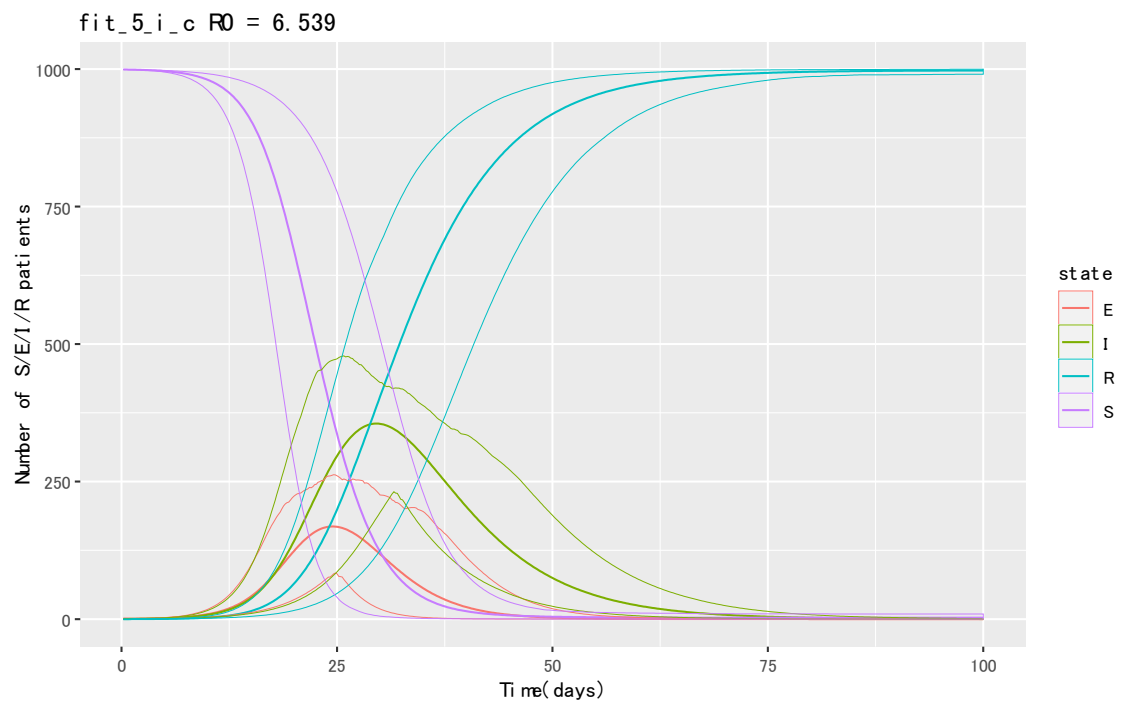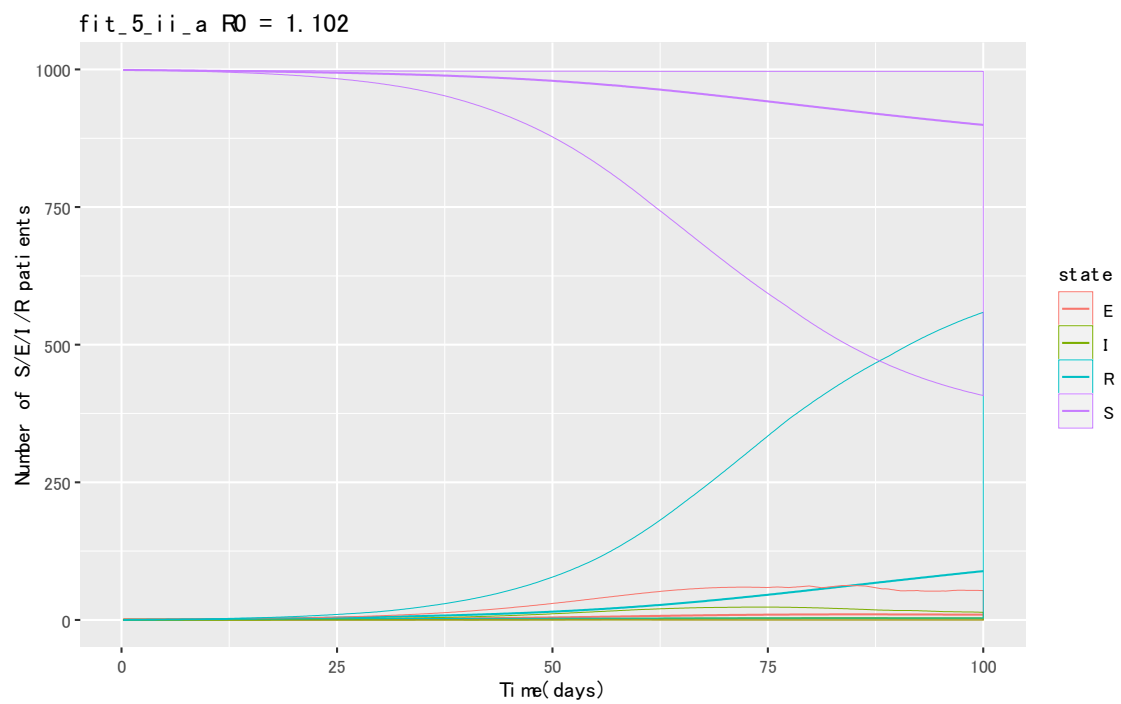

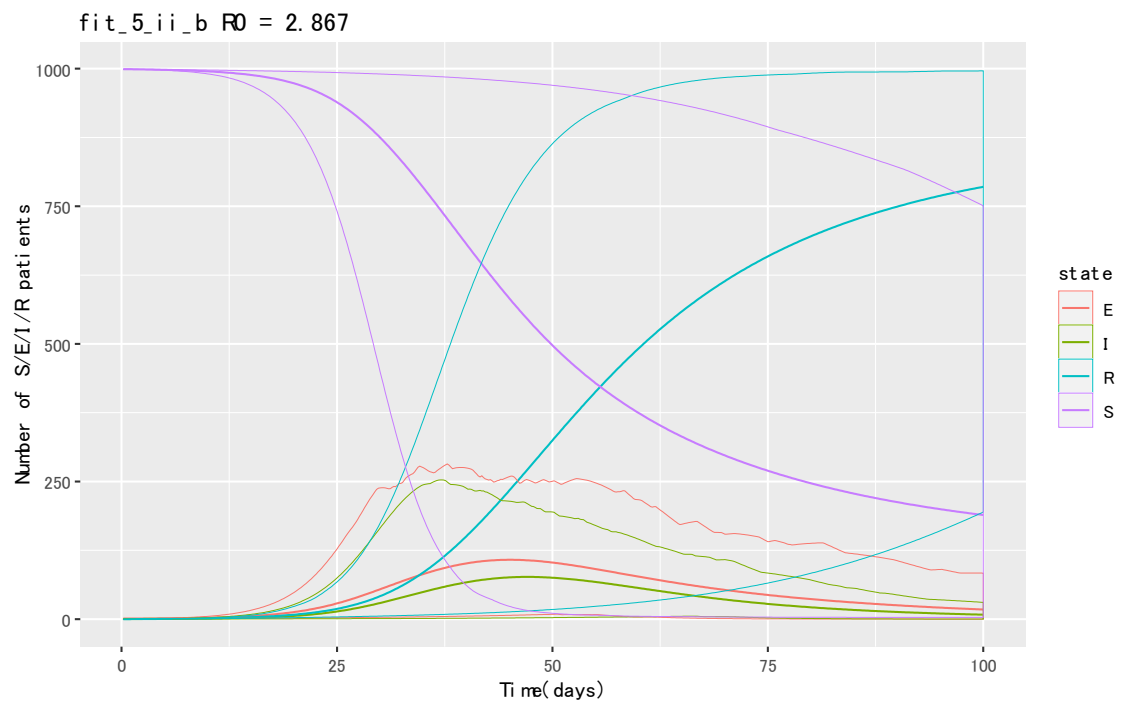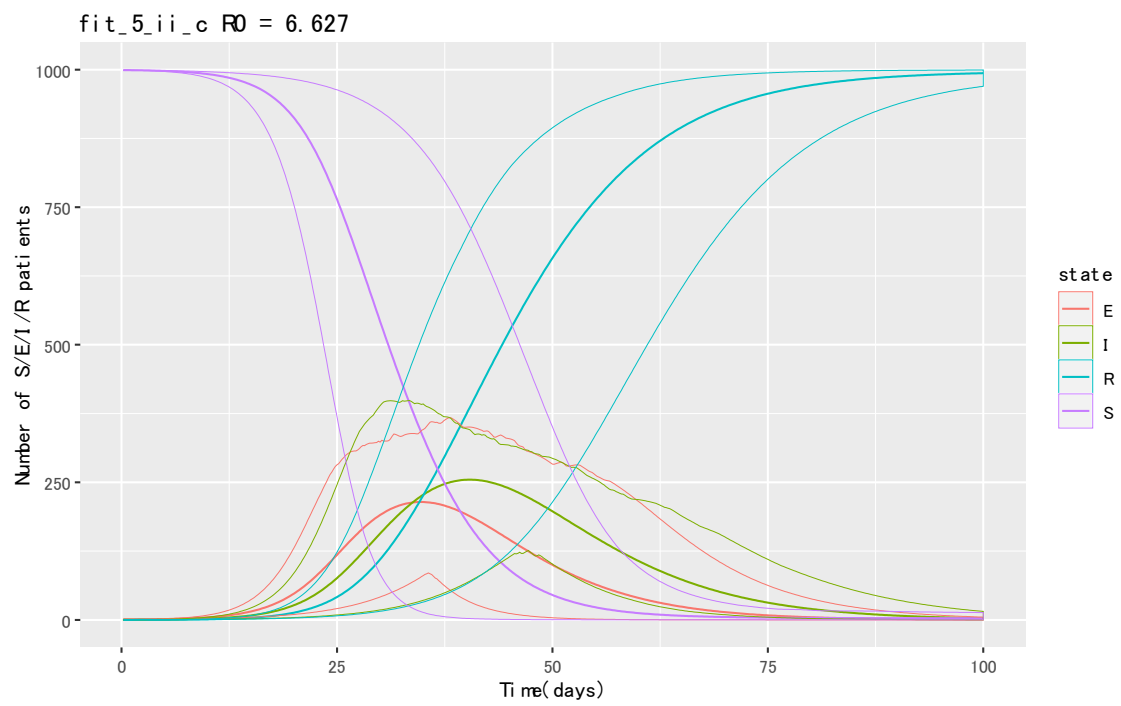

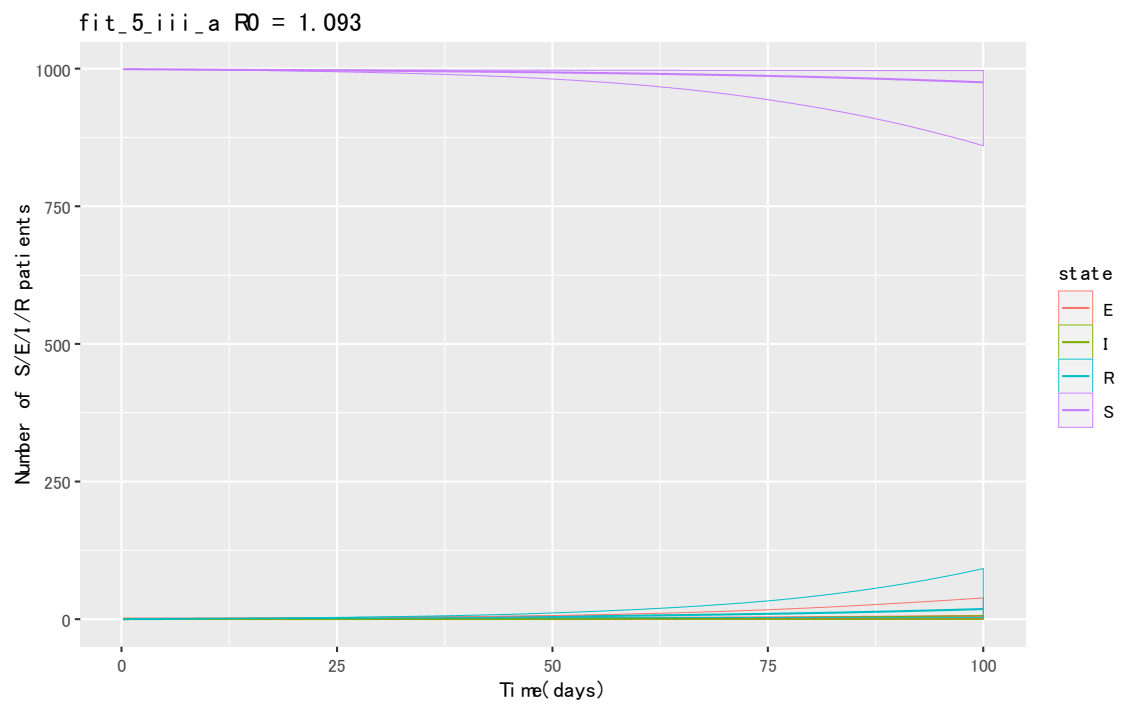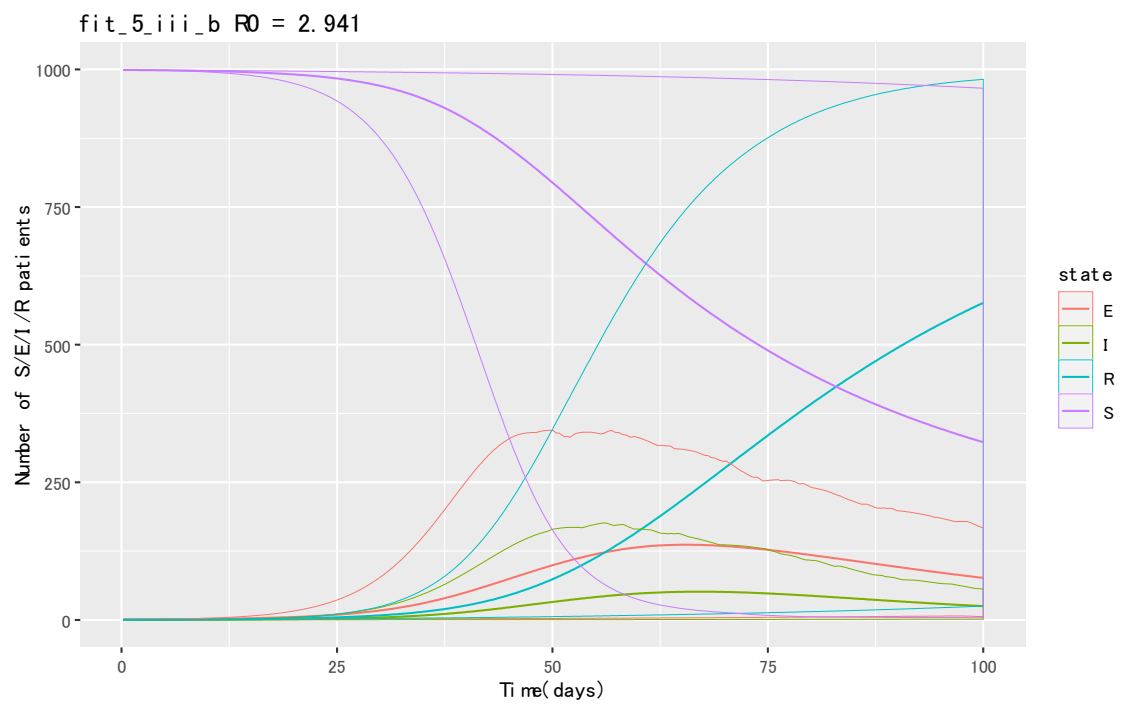

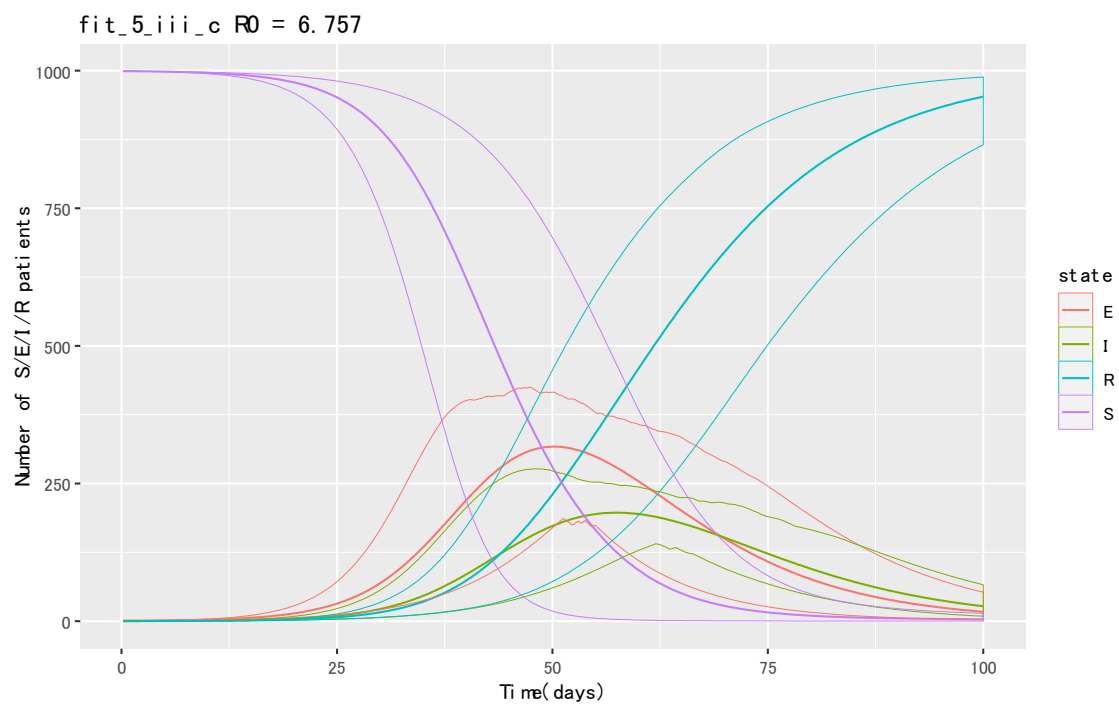

Supplement: Supplementary file 1 [file jcm-09-00944-s001.pdf]
